# Molecular Identification from AFM Images Using the IUPAC Nomenclature and Attribute Multimodal Recurrent Neural Networks

**DOI:** 10.1021/acsami.3c01550

**Published:** 2023-05-01

**Authors:** Jaime Carracedo-Cosme, Carlos Romero-Muñiz, Pablo Pou, Rubén Pérez

**Affiliations:** † Quasar Science Resources S.L., Camino de las Ceudas 2, E-28232 Las Rozas de Madrid, Spain; ‡ Departamento de Física Teórica de la Materia Condensada, Universidad Autónoma de Madrid, E-28049 Madrid, Spain; § Departamento de Física de la Materia Condensada, Universidad de Sevilla, P.O. Box 1065, 41080 Sevilla, Spain; ∥ Condensed Matter Physics Center (IFIMAC), Universidad Autónoma de Madrid, E-28049 Madrid, Spain

**Keywords:** atomic force microscopy, molecular identification, deep learning, neural network, image captioning, density functional theory

## Abstract

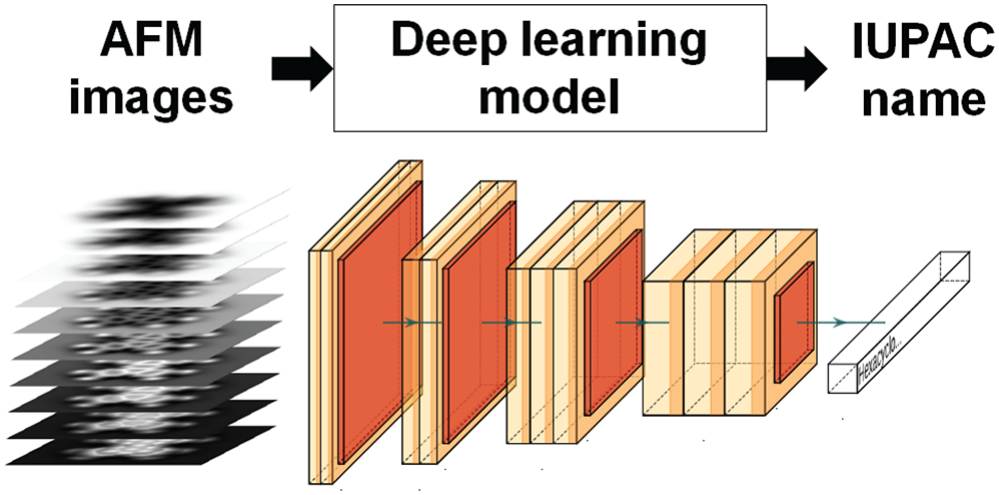

Spectroscopic methods—like nuclear magnetic resonance, mass spectrometry, X-ray diffraction, and UV/visible spectroscopies—applied to molecular ensembles have so far been the workhorse for molecular identification. Here, we propose a radically different chemical characterization approach, based on the ability of noncontact atomic force microscopy with metal tips functionalized with a CO molecule at the tip apex (referred as HR-AFM) to resolve the internal structure of individual molecules. Our work demonstrates that a stack of constant-height HR-AFM images carries enough chemical information for a complete identification (structure and composition) of quasiplanar organic molecules, and that this information can be retrieved using machine learning techniques that are able to disentangle the contribution of chemical composition, bond topology, and internal torsion of the molecule to the HR-AFM contrast. In particular, we exploit multimodal recurrent neural networks (M-RNN) that combine convolutional neural networks for image analysis and recurrent neural networks to deal with language processing, to formulate the molecular identification as an imaging captioning problem. The algorithm is trained using a data set—which contains almost 700,000 molecules and 165 million theoretical AFM images—to produce as final output the IUPAC name of the imaged molecule. Our extensive test with theoretical images and a few experimental ones shows the potential of deep learning algorithms in the automatic identification of molecular compounds by AFM. This achievement supports the development of on-surface synthesis and overcomes some limitations of spectroscopic methods in traditional solution-based synthesis.

## Introduction

Scanning probe microscopes have played a key role in the development of nanoscience as the fundamental tools for the local characterization and manipulation of matter with high spatial resolution. In particular, atomic force microscopy (AFM) operated in its frequency modulation mode allows the characterization and manipulation of all kinds of materials at the atomic scale.^1−3^ This is achieved measuring the change in the frequency of an oscillating tip due to its interaction with the sample. When the tip apex is functionalized with inert closed-shell atoms or molecules, particularly with a CO molecule, the resolution is dramatically enhanced, providing access to the inner structure of molecules.^4^ This outstanding contrast arises from the Pauli repulsion between the CO probe and the sample molecule^4,5^ modified by the electrostatic interaction between the potential created by the sample and the charge distribution associated with the oxygen lone pair at the probe.^6−8^ In addition, the flexibility of the molecular probe enhances the saddle lines of the total potential energy surface sensed by the CO.^9^ These high-resolution AFM (HR-AFM) capabilities have made it possible to visualize frontier orbitals^10^ and to determine bond order potentials^11^ and charge distributions^12,13^ and have opened the door to track and control on-surface chemical reactions.^14,15^

So far, the identification of molecular structure and composition relies heavily on spectroscopic methods like vibrational spectroscopy (i.e., Raman and IR), nuclear magnetic resonance (NMR) spectroscopy, or mass spectroscopy, together with X-ray based techniques. These techniques provide only averaged information extracted from macroscopic samples, and the chemical information collected in the acquired spectra is often difficult to interpret. In addition, X-ray-based methods are especially suited for crystalline samples and very limited in the case of organic molecules since H atoms cannot be detected by X-rays. Therefore, these traditional characterization techniques are not suitable for the study of the products and reaction intermediates of on-surface synthesis.^15^

At variance with those ensemble techniques, HR-AFM is able to address individual molecular entities, providing unique local information. This capability, together with the exquisite sensitivity of the AFM contrast to subtle changes in the molecular charge density, responsible for the impressive achievements described above,^16,17^ would suggest that HR-AFM could provide a completely different approach to molecular recognition, identifying not only the structure but also the chemical composition of a certain molecule exclusively by means of HR-AFM observations. However, this ultimate goal remains elusive. Molecules have been identified combining AFM with other experimental techniques like scanning tunneling microscopy (STM) or Kelvin probe force microscopy (KPFM), and with the support of theoretical simulations.^10,17−21^ Chemical identification by AFM of individual atoms at semiconductor surface alloys was achieved using reactive semiconductor apexes.^22^ In that case, the maximum attractive force between the tip apex and the probed atom on the sample carries information on the chemical species involved in the covalent interaction. However, the scenario is rather different when using tips functionalized with the inert CO molecules where the main AFM contrast source is the Pauli repulsion and the images are strongly affected by the probe relaxation. So far, the few attempts to discriminate atoms in molecules by HR-AFM have been based either on differences found in the tip–sample interaction decay at the molecular sites^6,23^ or on characteristic image features associated with the chemical properties of certain molecular components.^6,10,17,21,24−28^ For instance, sharper vertices are displayed for substitutional N atoms on hydrocarbon aromatic rings^6,23,24^ due to their lone pair. Furthermore, the decay of the CO–sample interaction over those substitutional N atoms is faster than over their neighboring C atoms.^6,23^ Halogen atoms can also be distinguished in AFM images thanks to their oval shape (associated with their σ-hole) and to the significantly stronger repulsion compared to atoms like nitrogen or carbon.^25^ However, even these atomic features depend significantly on the molecular structure^6,11^ and cannot be uniquely associated with a certain species but to its moiety in the molecule. Moreover, although the characteristic oval shape points immediately to the presence of a halogen, discriminating among the different chemical species has to rely on subtle details concerning the spatial extension of this feature and their variation with tip height. The huge variety of possible chemical environments and the need to consider the evolution with tip–sample distance of the AFM features render the molecular identification by a mere visual inspection by human eyes an impossible task.

Artificial intelligence (AI) techniques are precisely optimized to deal with this kind of subtle correlation and massive data. Deep learning (DL), with its outstanding ability to search for patterns, is nowadays routinely used to classify, interpret, describe, and analyze images,^29−34^ providing machines with capabilities hitherto unique to human beings or even surpassing them in some tasks.^35^ A DL model to achieve molecular identification faces two great challenges: (i) it has to be able to disentangle the contribution of the bonding topology, the chemical composition, and the internal torsion of the molecule to the AFM images, coping with the presence of experimental noise and tip asymmetries, and (ii) it should be able to generalize, learning from a training with a large but limited number of molecules to identify any possible organic molecule.

In this work, we solve these two challenges turning molecular identification into an image captioning problem: the description of the contents of an image through written words, a task where deep learning algorithms are especially appropriate. More specifically, we demonstrate that a stack of constant-height HR-AFM images (3D stack), covering the range of tip–sample distances where the interaction changes from attractive to repulsive, can be used as an input for a deep learning algorithm whose output is the International Union of Pure and Applied Chemistry (IUPAC) name of the target molecule. This algorithm learns during the training how to (i) recognize certain image features and their distance dependence in order to extract the chemical groups and their arrangement in the molecule from the 3D stack using a convolutional neural network (CNN) and (ii) use this chemical information to formulate following the rules of IUPAC nomenclature using a recurrent neural network (or Elman network) (RNN). As explained in detail below, we have used a two-step procedure, based on multimodal RNNs (M-RNNs), that is capable of identifying an unknown organic molecule from the AFM images.

The results presented below, based on a huge test with 816,000 3D stacks of AFM images belonging to 34,000 molecules that have not been used for the training, provide clear support to our two bold hypotheses: the significant chemical information contained in AFM images is enough to provide a complete molecular identification and can be retrieved using DL models. Our approach identifies with a 95% accuracy the chemical groups within the molecule. This aspect is really remarkable because not only is our model able to detect the presence of chemical functional groups, but it also determines how these groups are actually connected among them—given the IUPAC name—with a high accuracy. This is not the case of other well-established characterization techniques like vibrational spectroscopy or NMR, which only detect the presence of some constituent groups after a hard and complex assignment work of the spectra. In particular, our model predicts the exact IUPAC name in almost half of the tests and provides significant structural and compositional information in the rest of the cases, as shown by the high score—surpassing other applications in the literature—obtained with the Bilingual Evaluation Understudy (BLEU) algorithm,^36^ the most commonly applied metric to score the accuracy of language-involved models.

These achievements have great relevance for nanotechnology, where AFM is one of the key visualization and manipulation tools. It proves that, besides its already recognized ability to unveil the inner structure of molecules, the contrast observed in AFM images carries relevant chemical information, enough to allow the complete identification of the atomic species in the molecular composition. The analysis of particular cases in previous works hinted in this direction, but here we provide a clear answer to this question, which has remained elusive for many years. Ultimately, our work shows that AFM in combination with DL methods represents a powerful tool to obtain quantitative information about the spatial distribution of the electronic charge density in molecular systems. This has implications beyond molecular identification, not only for many nanotechnology applications that rely on subtle details of the molecular density, like self-assembly, but also for other relevant fields like the design and screening of more efficient catalysts, dyes for energy harvesting, and pharmaceutical drugs.

## Results

### Deep Learning Approach for Molecular Identification

The ultimate goal of the present work consists of designing and training a DL model that should be able to use as input experimental AFM images from an unknown molecule adsorbed on a certain substrate in order to produce an output that provides a complete molecular identification (structure and composition). Before considering the two challenges involved in extracting the chemical information from AFM images, there is a basic requirement that is common to all DL applications: the need of a very large data set to train the DL models. The amount of experimental data is certainly limited, but we should be able to rely on AFM simulation methods that are capable of accurately reproducing the observed contrast and its distance dependence in many systems. In a previous work,^37^ we have tested this hypothesis in a simple identification problem: the classification of a set of 60 organic molecular structures that include 10 different atomic species (C, H, N, P, O, S, F, Cl, Br, and I). We specifically designed a CNN, the neural network of choice for the analysis of images, and trained it with a large data set that includes 314,460 theoretical images of those molecules—calculated with the latest HR-AFM modeling approaches^6,38^—and only 540 images generated with a variational autoencoder from very few experimental images. Once trained, this CNN, using as input an AFM image of one of the molecules in the set—different from the ones used for the training—obtained almost perfect (99%) accuracy in the classification using simulated AFM images and very good accuracy (86%) for experimental AFM images. Notice that the correct identification of structures constituted by the same molecular entities is not a problem for machine learning algorithms as recently demonstrated for ionic hydrates.^39^

This proof-of-concept confirmed the feasibility of a molecular identification within a limited set using a model trained mostly with theoretical AFM images. Although encouraging, it is still very far from our final goal of a complete identification of an arbitrary molecule from AFM images. In particular, it clearly showed the need of a much richer training set. We have recently extended the available data sets of theoretical AFM images with the generation of Quasar Science Resources S.L.–Universidad Autónoma de Madrid–atomic force microscopy (QUAM–AFM),^40^ which aims to provide a solid basis for making results from DL applications to the AFM field reliable and reproducible.^41^ QUAM–AFM includes calculations for a collection of 686,000 molecules using 240 different combinations of AFM operation parameters (tip–molecule distance, cantilever oscillation amplitude, and tilting stiffness of the CO–metal bond), resulting in a total of 165 million images.^40^

Besides the need of large training data sets, the first intrinsic challenge of AFM-based molecular identification comes from the fact that the features of the AFM images are controlled by the charge density, which is uniquely related to the chemical nature and the position of the atoms within the molecule. The results in the AFM literature clearly show the exquisite sensitivity of AFM with CO tips to probe the molecular charge density, but a complete identification requires disentangling the contribution of the bonding topology, the chemical composition, and the internal torsion of the molecule to the AFM images through the molecular charge density. Alldritt et al.^42^ developed a CNN focused on the task of determining the molecular geometry. Results were excellent for the structure of quasiplanar molecules (with limited internal torsion), even using the algorithm directly with experimental images. For 3D structures, they were able to recover information for the positions of the atoms closer to the tip. However, the discrimination of functional groups produced nonconclusive results. At variance with this study, as we already mentioned above, a CNN^37^ was able to solve the classification problem for 60 essentially flat molecules with almost perfect accuracy, being able to identify, for example, the presence of a particular halogen (F, Cl, Br, or I) in molecular structures that, apart from this atom, were identical.

Tackling simultaneously the determination of the structure and the chemical composition remains an open problem. Even with the restriction to quasiplanar molecules, the clear success of the classification in ref (37) does not provide a general solution to the problem of molecular identification. The classification approach can only identify molecules included in the training data set. Given the rich complexity provided by organic chemistry, even using an extremely large data set—which already poses fantastic computational requirements (as the output vector has the dimension of the number of molecules in the data set)—the model would fail to classify many of the already known or possibly synthesized molecules of interest, which are not included in the training data set. Thus, the second intrinsic challenge is how to build a DL model that, trained with a large but limited number of molecules, is able to generalize and identify any possible organic molecule.

We have faced these two challenges and provided an answer to the two fundamental questions that they pose. Regarding the first challenge, we have considered the previous successes in the association of particular features in AFM images and their variations with tip–sample distance with certain chemical species, discussed above. Here, we propose using as input a stack: a collection of 10 constant-height AFM images, spanning (in intervals of 0.1 Å) the height range [2.8–3.7] Å above the molecular plane where the tip–sample interaction changes from slightly attractive to strongly repulsive in order to collect enough chemical information to determine the structure and composition of the molecular system.

To face the second challenge, we transform molecular identification into an image captioning problem: the description of the content of an image using language. Automatic image captioning has been a field of intensive research for deep learning techniques over the past several years.^43−46^ It has been recently and successfully used^47,48^ for optical chemical structure recognition,^49^ the translation of graphical molecular depictions into machine-readable formats. These works are able to predict the SMILES textual representation^50^ of a molecule from an image with its chemical structure depiction by using standard encoder–decoder^48^ or transformer^47^ models. In our case, we consider the stack of 10 constant-height HR-AFM images as the “image”, and the IUPAC name of the molecule as the description or caption. In the IUPAC nomenclature, a given name determines unambiguously the molecular composition and structure, so predicting the IUPAC name provides a complete identification of the molecule.

**Figure 1:**
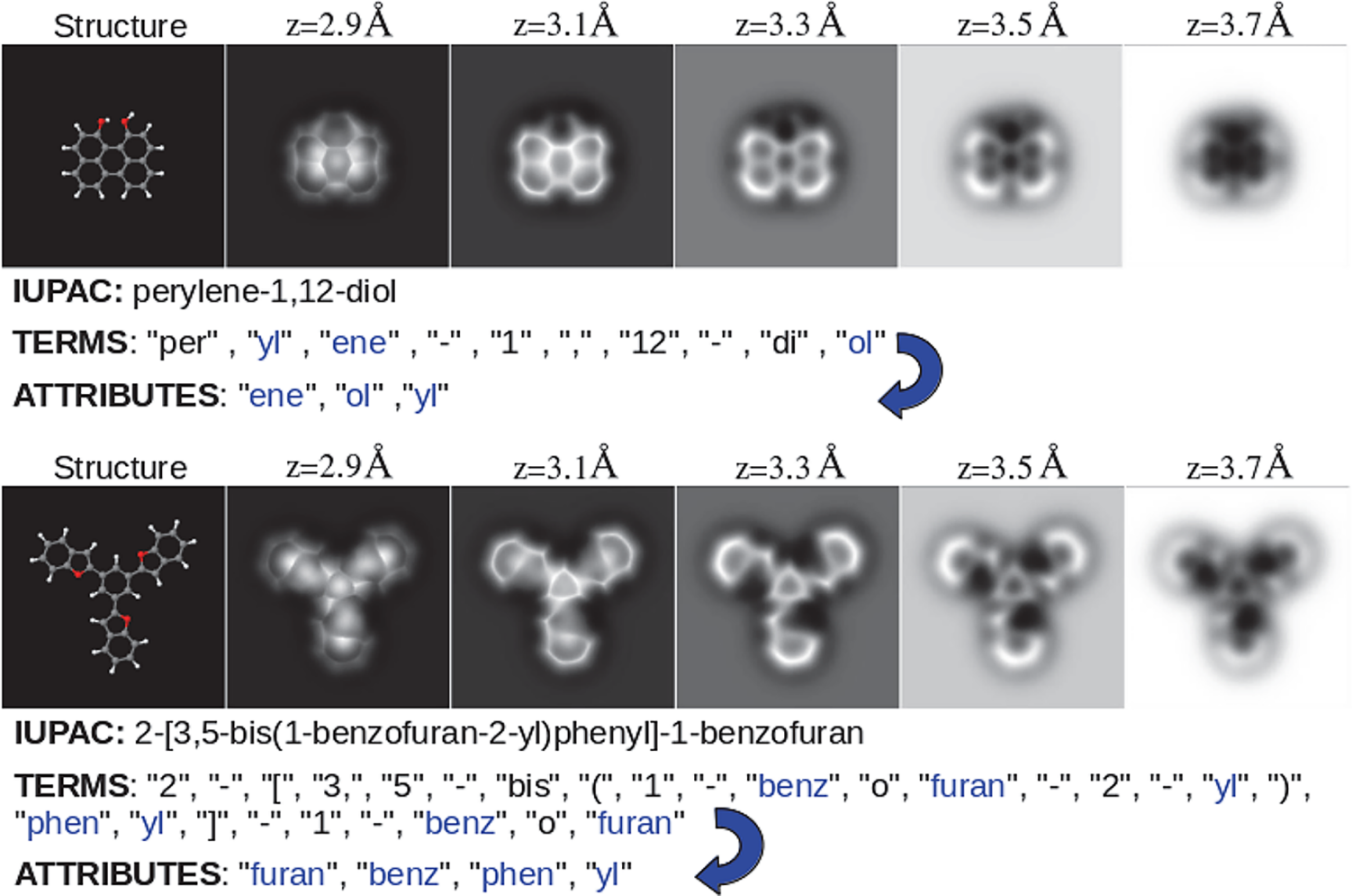
Two molecular structures with five of their associated AFM images at different tip–sample distances, the IUPAC name, the term decomposition of that name, and the associated attributes (a selection of 100 IUPAC terms that represent common functional groups, or chemical moieties; see the text). The top structure shows that the attributes are sorted by length and alphabetically, not by the position in which they appear in the term decomposition. The bottom structure shows that attributes appear once even if they are repeated in the term decomposition.

The IUPAC name is formed by combining *terms*: sets of letters, numbers, and symbols that are used to denote the functional groups, to define their structural position within the molecule, and to specify their connections.^51^ The *terms*, taken from a hierarchical keyword list, play the role of “words” and together with the “syntax”, the systematic rules to assemble additive names, define the language that we are going to use to describe the content of our AFM images. We name as *attributes* the elements in the subset of *terms* that identify the functional groups or moieties (see Table S1 and the Methods section). Figure 1 illustrates how the combinations of these terms generate the IUPAC names for two molecules and identifies the *attributes* in those names.

**Figure 2:**
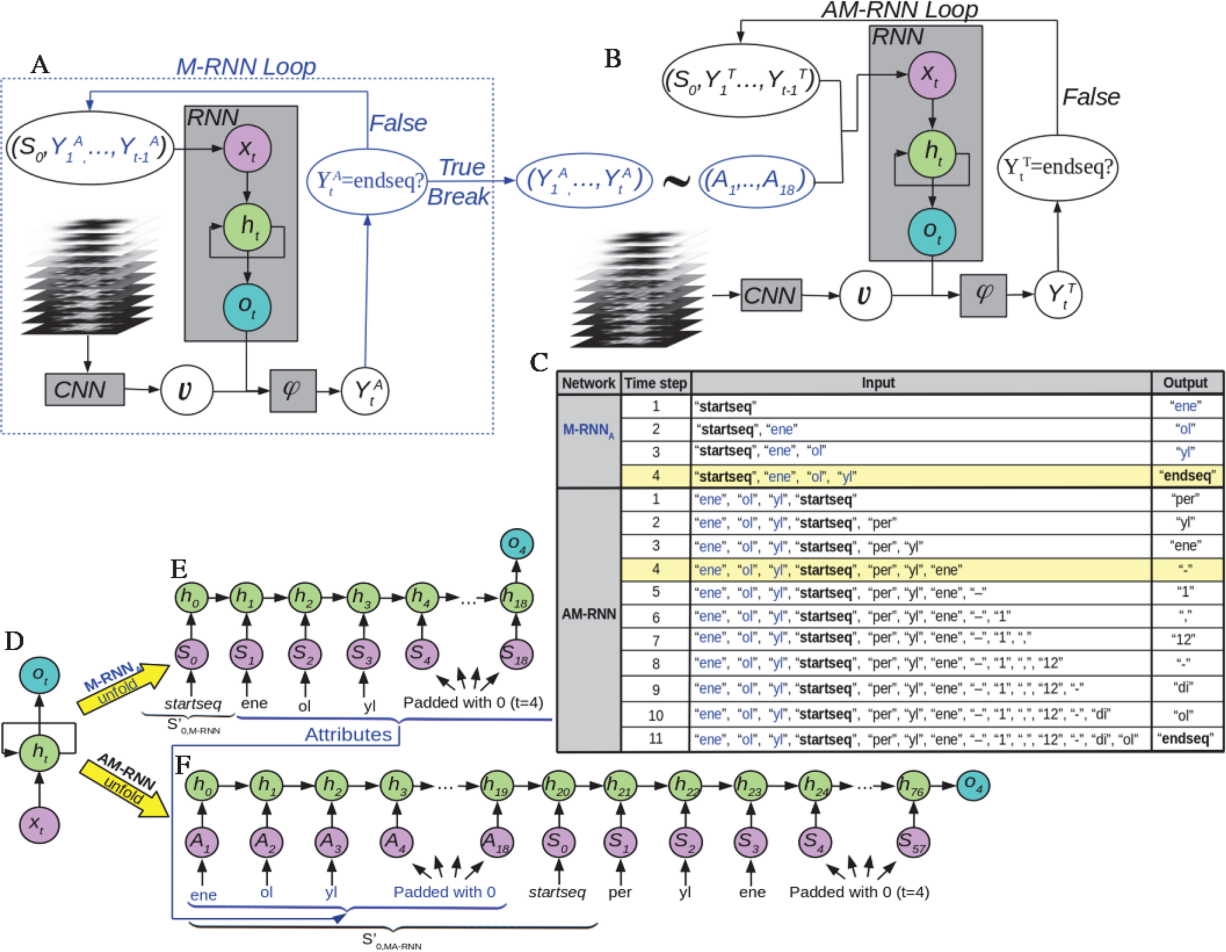
The architecture proposed for molecular identification through the IUPAC name is a composition of two networks [Multimodal Recurrent Neural Network for attribute prediction (M-RNN_*A*_) and Atribute Multimodal Recurrent Neural Network (AM-RNN)] whose data flow is shown in panels A and B. The gray square boxes represent each component of the models: a convolutional neural network CNN, a recurrent neural network RNN, and the multimodal component φ. A detailed description of the structure and role of the CNN, RNN, and multimodal φ components can be found in Figure S1 and Sections S2 and S3. The arrows indicate the data flow within the model. M-RNN_*A*_ predicts an attribute at each time step until the loop is broken with the *endseq* token (blue-line printed), whereas the AM-RNN predicts the sorted terms that give rise to the IUPAC name. Panel C shows the inputs and outputs at each time step predicted by the M-RNN_*A*_ and AM-RNN networks from a 3D image stack corresponding to the perylene-1,12-diol molecule (see Figure 1). (D–F) Representation of the RNN, in the same format used in panels A and B, corresponding to the fourth time step in M-RNN_*A*_ (E) and AM-RNN (F) for the perylene-1,12-diol molecule. This figure highlights the fact that the state of the RNN, in particular, the recurrent layer, depends on the previous predictions.

Most of the current methods for automatic image captioning have two key components: (i) a CNN—a neural network (NN) with convolutional kernels as processing units—that represents the high-level features of the input images in a reduced dimensional space, and (ii) an RNN^52^—an NN whose units are complex structures that have an inner state that stores the temporal context of a time series—that deals with language processing and predicts a single word at each time step.^53−55^ In our implementation, we focus on the well-known Multimodal Recurrent Neural Network (M-RNN), which integrates three components (see Figure 2A and Figure S1). Besides the CNN and RNN, there is a multimodal (φ) component, which concatenates the CNN and RNN outputs in a single vector and uses fully connected layers to search for relations among the components of this vector (representing the high level features extracted by the CNN and the predictions of the CNN) to generate the output of the model. At each time step, our model has to predict segments of the molecule’s IUPAC name (see Figure 1).

Our first attempts based on feeding an M-RNN with a stack of AFM images provided poor results predicting the IUPAC names. For this reason, we decomposed the problem into two parts and developed an architecture composed of two M-RNNs (see Figure 2A,B). M-RNN_*A*_ (Figure 2A) uses as input the stack of AFM images and predicts the *attributes*, the main chemical groups that compose the molecule. The second network, named AM-RNN, takes as inputs both the AFM image stack and the attribute list with the aim of ordering them and completes the whole IUPAC name of the molecule with the remaining terms. Figure 2C shows the inputs and outputs at each time step predicted by the M-RNN_*A*_ and AM-RNN networks from a 3D image stack corresponding to the perylene-1,12-diol molecule. Although both AM-RNN and M-RNN_*A*_ are based on the standard M-RNN,^43^ we introduce substantial modifications in each component (see the Methods section).

The QUAM–AFM^40^ data set has been used to train and test the networks (see the Methods section). A description of each layer and the training strategy, far from trivial when combining a CNN and an RNN, can be found in Sections S2 and S3, respectively. We have to stress here that, regarding the design and the training of the DL models, we have always kept in mind that our final goal is to be able to identify molecular systems from experimental AFM images. To this end we have used during the training stacks of images corresponding to 24 different combinations of AFM operation conditions (cantilever oscillation amplitude and tilting stiffness of the CO–metal bond) and applied an Imaged Data Generator (IDG) to take into account deformations in the images (due to slight asymmetries of the CO tip or to experimental noise) (see Section S3). Furthermore, we have also considered this in the design of the model, including some dropout layers in the CNN, the RNN, and the multimodal component (as described in Section S2), to prevent the model from overspecializing in the theoretical images.

### Assessment of the Model

**Figure 3:**
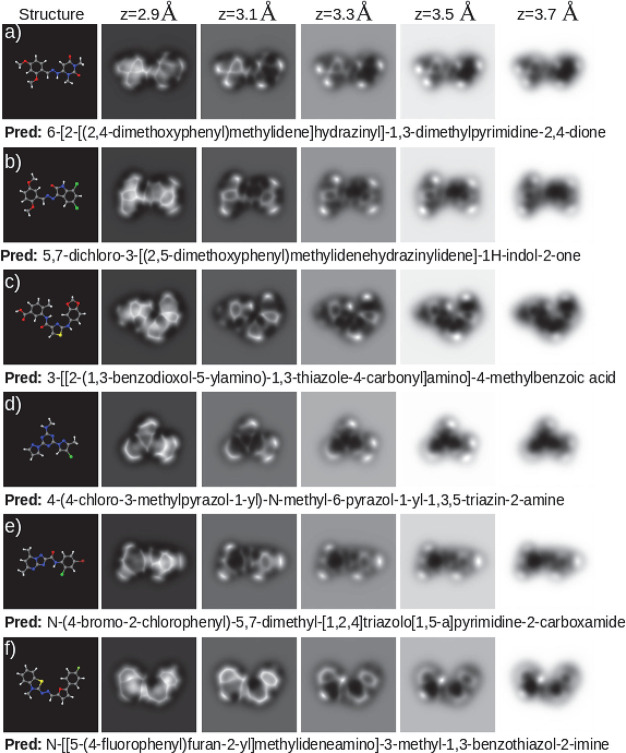
Set of perfect predictions (4-gram scores of 1.00). Each panel shows the molecular structure on the left, the AFM images at various tip–sample distances on the right, and the prediction, which matches exactly the ground truth, below the images.

We have benchmarked the model illustrated in Figure 2 by testing the trained networks with the 34,000 molecule test set, corresponding to a total of 816,000 testing inputs from QUAM–AFM associated with 24 different combinations of the AFM simulation parameters. M-RNN_*A*_ predicts the correct list of functional groups in the molecule in 95% of the cases. This result answers one of the more challenging open questions in the field:^6,10,23^ it demonstrates that the 3D HR-AFM data obtained with CO-terminated apexes carries information on the chemical species present on the molecules, at least on the simulated image sets. The IUPAC names predicted by the AM-RNN network are identical to the annotations for 43% of the molecules. Taken into account the complexity of the problem, we can consider this as a good result. Notice that each match means that the model has identified from the images, without any error, all the molecular moieties, and it has also provided the exact IUPAC name, character by character, as shown in Figure 3. Our model is able to identify planar hydrocarbons, either cyclic or aliphatic, but also more complex structures such as those including nitrogen or oxygen atoms that, due to their fast charge density decay,^6^ usually appear on the images as faint features (see for example Figure 3). Halogens, characterized on the images by oval features whose size and intensity are proportional to their σ-hole strength,^25^ can also be correctly labeled (Figure 3b,d,e). The model can even recognize the presence of the fluorine element, which does not induce a σ-hole and, when bonded to a carbon atom, produces an AFM fingerprint that is very similar to that of a carbonyl group (compare Figure 3e with Figure 3f). More surprisingly, hydrogen positions are often guessed, which is striking since hydrogen atoms bonded to sp^2^ carbon atoms are hardly detected by the HR-AFM due to their negligible charge density.^28,56^ Thus, many kinds of molecules, over half of our test set, including those showing nontrivial behaviors, have been correctly recognized by our model.

**Figure 4:**
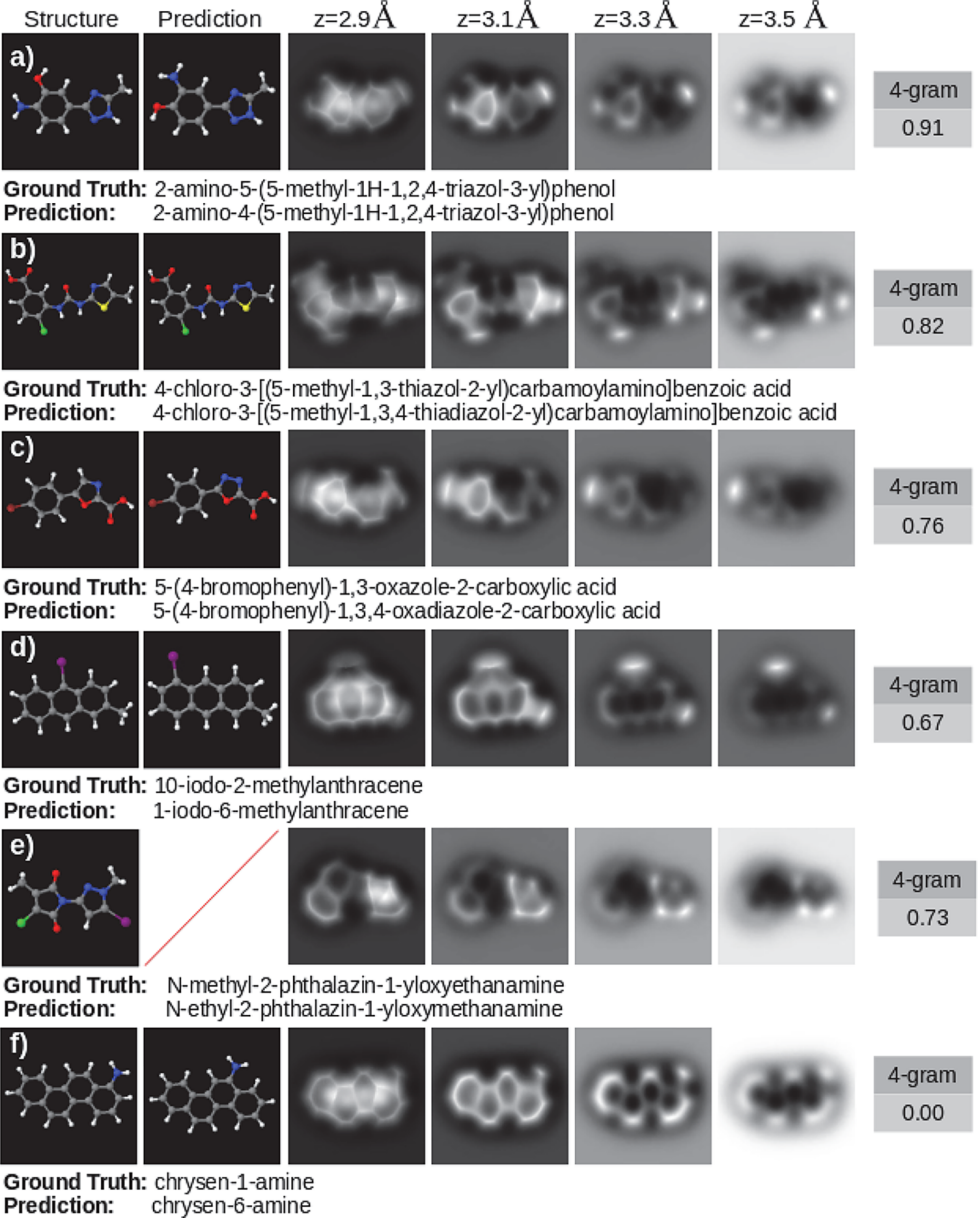
Examples of incorrect predictions, reflecting how the evaluation algorithm penalizes errors. Each panel shows, from left to right, the simulated structure, the structure that matches the model prediction (where it exists), a set of AFM images at various tip–sample distances, and the 4-gram score. Below each panel is the IUPAC name of the molecule (ground truth) and the prediction performed by the model (prediction).

However, this statistic does not reflect the real accuracy of the model. A deeper analysis of the results shows that its quality and usefulness is much higher than the naked figure of 43% could indicate. Figure 4 shows that, even in those cases where the prediction is not correct, the majority of the examples still provide valuable information about the molecule. In order to quantify the accuracy of the prediction, we apply the *n*-grams of BLEU^36^ (see Figure 4). This method, commonly used for assessing accuracy in natural language processing (NLP) problems, calculates the accuracy based on *n-grams* of *terms* between predicted and reference sequences. An *n-gram* scores each prediction by comparing the sorted *n*-word groups appearing in the prediction with respect to the references. In our scenario, the comparison is with one single reference (ground truth), so it compares the common groups of *n terms* that appear in both the prediction and the reference (for example, perylene-1,12-diol, 4-gram reference groups include: “per, yl, ene, −”, “yl, ene, −, 1”, “ene, −, 1, ,”, etc.).

First, we apply the BLEU metric to assess the accuracy of the M-RNN_*A*_, the one that predicts the *attributes*, i.e., the molecular moieties. A perfect match on the 1-grams means that every *attribute* in the reference appears in the prediction and that the prediction does not contain any other *attributes*. Our model scores 0.95 under this assumption. This very high mark confirms that this network does recognize the molecular components in 95% of the cases.

For the assessment of the overall prediction of the model, we propose the cumulative 4-gram, a common metric for the evaluation of linguistic predictions. This metric weights the scores obtained with the 1,2,3,4-grams and also performs a product with a function that penalizes the different lengths between prediction and reference. BLEU scores (see Table 1) reveal that AM-RNN also performs exceptionally well. Note that, in this case, the 1-gram shows the set of *terms* that are in both prediction and reference. That is, despite not providing the correct formulation, the model is able to predict 88% of the *terms* that the name contains, in agreement with the prediction capability shown by our first M-RNN_*A*_, and indicating a great deal of chemical information about the molecule. In addition, AM-RNN scores 0.76 in the evaluation with the cumulative 4-gram, assessing large segments of the IUPAC name. Figure 4 puts the accuracy of the model based on this assessment in context with a set of examples with different scores. Note that Figure 4f shows a frequently occurring case where, by applying a metric developed to assess translation in longer texts with several references, mistakes in predictions composed of only a few *terms* are overly penalized. Table 2 provides a systematic study of this limitation of the metric, showing an analysis of the score obtained by splitting the test set according to the number of terms into which the corresponding IUPAC name decomposes. The accuracy of the model is worse in molecules whose term decomposition is shorter. The reason for this seemingly contradictory fact is that the cumulative 4-gram metric penalizes more for errors in short chains. As shorter strings contain fewer subgroups of 4 terms, the 4-gram scoring method penalizes an error in a smaller chain more heavily than in a longer one (as shown in Figure 4a,f).

**Table 1:** BLEU Cumulative *n*-Gram Scores Obtained with AM-RNN^a^

metric	1-gram	2-gram	3-gram	4-gram
score	0.88	0.84	0.79	0.76

a The test has been performed on 816,000 inputs (3D stacks of constant-height HR-AFM images) taken from the QUAM–AFM data set, corresponding to a set of 34,000 molecular structures simulated with 24 different combinations of AFM operation parameters.

**Table 2:** Score with the BLEU Cumulative 4-Gram Metric Based on the Characteristics of the Molecules and Their Annotations^a^

Number of Terms						
term decomposition	0–10	10–20	20–30	30–40	40–50	50–60
4-gram score	0.59	0.73	0.78	0.79	0.75	0.66

Atom Height Difference				
distance	<0.5 Å	<1.0 Å	<1.5 Å	≥1.5 Å
4-gram score	0.79	0.62	0.62	0.50

a The top section divides the scores into subsets based on the length of the string of *terms* into which the IUPAC name is broken down. The bottom section divides the test set score into subsets based on the maximum difference in height among atoms in the molecule (excluding hydrogens).

Comparing the predictions with the references on a term-by-term basis, we find that 25.1% of the errors are due to misclassification of one number term with another number, i.e., misplacing a group of atoms, and 17.1%, 4.8%, 4.7%, and 2.7% of the errors are due to a misclassification of the “–”, “(” or “)”, “yl”, and “[” or “]” *terms*, respectively. Therefore, almost half of the errors made are located in the prediction of characters more related to the chemical formulation than to the information extracted from the images. Moreover, we must point out the fact that when the model predicts incorrectly, it sometimes generates IUPAC names that do not correspond to any molecule (see Figure 4e). These results indicate that it is not the capability of our model to recognize the molecules but the ability of the RNN component to properly write the name that is limiting the success rate. This conclusion is consistent with a recent work where automated IUPAC name translation from the SMILES nomenclature,^50^ which completely characterizes the structure and composition of a molecule, is done by an RNN,^57^ obtaining a BLEU 4-gram score of just 0.86.

Deep learning architectures are developed based on human intuition to improve the accuracy of the model. However, it is difficult to analyze in detail why the model succeeds or fails. When representing the input *terms* in the RNN component, we apply a word embedding that is trained with the rest of the model (see Figures S2 and S3). Previous research has shown that representations in this space capture the semantic meaning of words and establish algebraic relationships between them.^58−60^ It is truly remarkable to see that these results have been transferred to the formulation, grouping the *terms* according to their semantic meaning or according to the interactions described by the image stacks. We have verified this by projecting each of the *terms* into the 32-dimensional embedding space belonging to the AM-RNN, defining an L2 norm and computing the distances between the *terms*. These results show that *terms* with similar semantic meaning are close together (see Figure S9). For example, the closest *terms* to brom are chlor, fluor, and iod, or the *terms* closest to nona are octa, deca, undeca, and dodeca. This also reflects in the fact that the *terms* that the model most commonly gets wrong are the closest ones, such as the errors in the prediction of the numbers that place atomic groups in specific positions (see Figure 4a,d,f), or the mistaking of one halogen for another. In other words, the erroneous terms have, in general, a similar semantic meaning.

Nonplanar structures are a challenge for AFM-based molecular identification. Table 2 shows an analysis of the score obtained by splitting the test set according to different ranges of molecular torsion. In line with the conclusions reached in ref (42), our model has a hard job to fully reveal the structure of molecules whose height difference between atoms exceeds 1.5 Å. This is an expected result as the microscope is highly sensitive to small variations on the probe–sample separation, and the interaction becomes highly repulsive on a distance range of 50–100 pm, inducing large CO tilting and image distortions. This makes it very difficult to get a proper signal from lower atoms on molecules with nonplanar configurations. We have tested our model by randomly selecting four of the nonplanar structures whose prediction scores an arithmetic mean of 0.40 in the cumulative prediction of the 4-gram. We force them to acquire a flat structure, and then, we run the test again (see Figures S6 and S7). Prediction scores improve in the range 0.2–0.55, resulting in a new mean cumulative 4-gram of 0.73. This improvement represents semantically going from a prediction that barely provides any useful information about the molecule to one that in many cases gets it absolutely right. Thus, while the model already scores very high in the test with simulated images of gas-phase molecules, the performance would definitely improve with the flatter configurations expected for the adsorbed molecules measured in the experimental HR-AFM images. In this regard, a recent work^61^ has shown how the limitations of AFM with bulky molecules can be overcome with the combination of AFM imaging with Bayesian inference and DFT calculations in order to determine the adsorption configurations for a known molecule. Future work should explore whether a combination of this strategy with our models is able to extend the molecular identification to highly corrugated structures.

## Discussion

The results presented so far show that the stacks of 3D frequency shift images contain information not only on the structure of the molecules but also regarding their chemical composition. This information can be extracted by deep learning techniques, which, additionally, are able to provide the IUPAC name of the imaged molecules with a high success rate. Our combination of two M-RNNs is able to correctly recognize the molecule in many cases, even in those where it is difficult to discern between similar functional groups—as fluorine terminations with either carbonyl or even −H terminations—or in image stacks where some moieties provide very subtle signals (see Figure 3). Some mistakes do appear from the chemical recognition point of view, especially in those molecules showing significant nonplanar configurations where the performance is lower (see Figures S6 and S7 together with Table 2). However, apart from these fundamental drawbacks, most of the errors in the predictions are related to the spelling of IUPAC names: that is, misplacement of functional groups or the incorrect use of parentheses, square brackets, or hyphen characters, etc. It seems that these errors are frequent for RNNs dealing with the IUPAC nomenclature.^57^

At this stage, it is worth considering if other DL architectures or alternative chemical nomenclatures could improve the molecular identification based on HR-AFM images. We have already pointed out that, leaving out the additional problem of extracting the chemical information from the images, an RNN only achieves a BLEU 4-gram score of 0.86 when translating from the SMILES to the IUPAC name.^57^ Nomenclature translation has been addressed with architectures based on the novel transformer networks,^62^ obtaining a practically perfect accuracy.^63,64^ Also, automatic recognition of molecular graphical depictions is able to correctly translate them to their SMILES representation with a 88% or 96% accuracy by using either a standard encoder–decoder^48^ or a transformer^47^ network. However, in our work we face a different problem since we deal with identification from AFM images instead of either molecular depictions, which contains all the chemical information needed to name a molecule, or translation between nomenclatures. Furthermore, the application of transformers to the identification from AFM images is not straightforward. First, tokenization must be consistent, and each term must have a chemical meaning so that the embedding layers learn a meaningful information representation (see Figure S9). This point has only been considered in ref (63). More importantly, our method achieves high accuracy due to the initial attribute detection, forcing us to develop an architecture that is, in principle, incompatible with transformers, which are based on encoder–decoder networks.

Regarding other nomenclature systems for describing organic molecules, besides the already cited SMILES,^50^ there are other proposals such as InChI^65^ or SELFIES,^66^ whose textual identifiers use the name of the atoms and bond connectivity. These systems miss relevant chemical information that is not provided by describing the molecule as a set of individual atoms rather than as moieties made up of atoms. Unlike these systems, the IUPAC nomenclature is focused on the classification of functional groups, an approach consistent with the characteristics shown by the AFM image features, that reflects in our proposal of a dual architecture composed by M-RNN_*A*_ and AM-RNN. The SELFIES nomenclature establishes a robust representation of graphs with semantic constraints, solving some problems that arise in computer writing with other nomenclatures. However, the atom-based description would force an approach without attribute prediction, which is the key to obtaining a high accuracy with our model. Hence, it seems to be a trade-off between the limitations and improvements offered by these nomenclatures, suggesting that a dramatic improvement in performance is not expected, although further work is needed in order to reach a final conclusion.

Finally, we should recall that, although our final goal is a method to identify the structure and composition of molecules from their experimental HR-AFM images, our analysis so far has been based on simulated images. In ref (37), we showed that the experimental images contain features that are not reflected in the theoretical simulations. Data augmentation has been applied during the training (see Subsections S3.2 and S3.4) to capture these effects, and specific features have been included in the model (like the dropout layers in the RNN; see Figure S1) to prevent it from specializing too much with theoretical images. Although limited by the scarcity of experimental results suitable to apply our methodology, the tests have provided very promising results. We have selected constant-height AFM images of dibenzothiophene and 2-iodotriphenylene from refs (27) and (67), corresponding to 10 different tip–sample distances, covering a height range of 100 pm for dibenzothiophene (identical to the one spanned by the 3D stacks of theoretical images used to train our model) and 72 pm for 2-iodotriphenylene (see Section S5 for details). Despite the strong noise in the images and the white lines crossing the images diagonally (see Figure S8), the prediction of dibenzothiophene is perfect, scoring 1.00 on the 4-gram, whereas for 2-iodotriphenylene the model predicts “2iodtriphenylene”, missing a hyphen but providing all the relevant chemical information. Despite these good results, a larger, systematic analysis with proper experimental data is necessary to further address the accuracy of our model.

## Conclusions

In this work, we have shown how deep learning models, trained with the simulated HR-AFM 3D image stacks for 678,000 molecules included in the QUAM–AFM data set, are able to perform full chemical–structural identification of molecules. Motivated by the unfeasibility of defining a classification in the usual sense of AI, we turned the problem into an image captioning problem. Thus, instead of aiming to have a model that knows every atomic structure, we endow it with the ability to formulate. As a result the model is able to not only identify images that have not been previously shown to it but also predict the IUPAC name of these unknown structures. We have devised a two-step procedure involving the combination of two M-RNNs. In a first step, the M-RNN_*A*_ identifies the attributes, the most relevant functional groups present in the molecule. This initial step is already of importance because the algorithm provides useful information about the chemical characteristics of the molecule. In a second step, the AM-RNN, whose inputs arise from the M-RNN_*A*_, sorts the information on the functional groups and adds extra characters (connectors, position labels, other tags, etc.) and the remaining functional groups that are not part of the *attributes* set. That is, the AM-RNN assigns the positions of the functional groups, completes the remaining terms, and writes down the final IUPAC name of the molecule.

We have tested the model on a set of 816,000 3D stacks of HR-AFM images belonging to 34,000 molecules that have not been shown before to the network. The predictions for the IUPAC names are exactly the same with respect to the reference in QUAM–AFM, character by character, in a striking 43% of the cases. To further asses the usefulness of the wrong predictions by the model, we apply the metrics defined by the BLEU *n*-gram. The accuracy of the *attribute* prediction assessed with the 1-gram scores 0.95: our approach correctly identifies all the functional groups in the molecule in 95% of the cases, demonstrating that the 3D image stacks carry key chemical information. The overall accuracy of the model is determined with the cumulative 4-gram, scoring 0.76. This high value means that, even when the model does not achieve a perfect prediction, it provides valuable chemical insight, leading to a correct IUPAC name of a similar molecule in the vast majority of the cases. The ability of machine learning models to provide relevant information from HR-AFM images is further supported by alternative approaches based on CNNs to predict accurate electrostatic fields^68^ and on graph neural networks (GNNs) to extract molecular graphs.^69^ The accuracy obtained in the extensive test with theoretical images, together with the results from few experimental examples taken from the literature, shows the potential of our deep learning approach trained with theoretical results to become a powerful tool for molecular identification from experimental HR-AFM images.

## Methods

### QUAM–AFM: Structures and AFM Simulations

One of the main challenges to automate the molecular identification through AFM imaging arises from the limited availability of data to fit the parameters of deep learning models. We use QUAM–AFM,^40^ a data set of 165 million AFM images theoretically generated from 686,000 isolated molecules. Although the general operation of the HR-AFM is common to all instruments, operational parameter settings (cantilever oscillation amplitude, tip–sample distance, CO tilt stiffness) lead to variations in the contrast observed on the resulting images. The value of the first two can be adjusted by modifying the microscope settings to enhance different features of the image. However, the latter depends on the nature of the tip, i.e., the differences in the attachment of the CO molecule to the metal tip that have been consistently observed and characterized in experiments.^38,70^ In order to cover the widest range of variants in the AFM images, six different values for the cantilever oscillation amplitude, four for the tilt stiffness of the CO molecule, and 10 tip–sample distances were used to generate QUAM–AFM, resulting in a total of 240 simulations from each structure. We use the stack of 10 images resulting from the different tip–sample distances in a single input and the 24 parameter combinations as a data augmentation technique. That is, we feed the network with different image stacks randomly selected from the combinations of simulation parameters in each of the epochs for each of the molecules.

### IUPAC Tokenization

Deep learning has already proven to have an extraordinary capacity to analyze data. This capacity is such that, in many cases, the biggest problem to be solved lies in defining an appropriate descriptor rather than in improving the existing analysis capacity. This is the case for AFM images, where the complexity to design the output of a model is due to the existence of infinite molecular structures. To establish a model output that is unambiguous, uniform, and consistent for the terminology of chemical compounds, we have adopted the IUPAC nomenclature. Then, we have turned the standard classification problem^37^ for a finite number of molecular structures into an image captioning task, developing a model that manages to formulate the IUPAC name of each molecule.

Most image captioning techniques to describe images through language consist of a loop that predicts a new word at each iteration (time step). Our goal is to transfer this idea to the identification of AFM images through the IUPAC formulation. Therefore, instead of predicting words at each time step, our model has to predict segments of the molecule’s IUPAC name (see Figure 1). That is, the set of tokens used to decompose each name are sets of letters, numbers, and symbols that we call *terms* and are used by the IUPAC nomenclature to denote functional groups, to assemble additive names, or to specify connections. Different combinations of these terms generate IUPAC names for the molecules, as exemplified in Figure 1.

A systematic split of the IUPAC names in QUAM–AFM reveals that some of the terms have a very small representation, not enough to train an NN. We have discarded those that are repeated less than 100 times in QUAM–AFM, retaining a total of 199 terms (see Table S1). Consequently, we have also removed the molecules that have any of these terms in their IUPAC name. In addition, we have dropped the molecules whose term decomposition has a length longer than 57, as there is not enough representation of such names in QUAM–AFM. Even so, the set of annotations still contains 678,000 molecules, that we have split into training, validation, and test subsets with 620,000, 24,000, and 34,000 structures, respectively.

Our first attempts based on feeding a single model with a stack of AFM images provide poor results predicting the IUPAC nomenclature. For this reason, we decompose the problem into two parts and assign each objective to a different NN (see Figures 1 and 2 and the next section for a detailed description). We define the *attributes* as a 100-element subset of the IUPAC terms (see Table S1) which mainly describes the most common functional groups in organic chemistry and, thus, are repeated a minimum number of times. The first network, named M-RNN_*A*_, uses as input the stack of AFM images, and its aim is to extract the attributes, predicting the main functional groups of the molecule (see Figures 1 and 2). The second network, named AM-RNN, takes as inputs both the AFM image stack and the attribute list with the aim of ordering them and completing the whole IUPAC name of the molecule with the remaining terms which are not considered attributes (see Figure 2B).

M-RNN_*A*_ reports information neither on the order nor the number of times that the *attribute* appears in the formulation. However, this first prediction plays a key role in the performance of the model. Unlike most of the NLP challenges, the IUPAC name completely identifies the structure and composition of the molecule. Thus, a prior identification of the main functional groups not only releases the CNN component of the AM-RNN from the goal of identifying these moieties but also, more importantly, almost halves the number of possible predictions of the AM-RNN. By feeding the AM-RNN with the attributes that are present in the IUPAC name (predicted by the M-RNN_*A*_), we are also effectively excluding the large number of them that do not form part of it. This is an extremely simple relationship that the network learns and that significantly improves its performance.

### Multimodal and Attribute Multimodal Recurrent Neural Networks (M-RNN_A_ and AM-RNN)

The standard approach for image captioning is based on an architecture that integrates a CNN and an RNN.^43,71^ Here, we focus on the well-known M-RNN, which integrates three components (see Figure S1). The CNN encodes the input image into a high-level feature vector whereas the RNN component has two key objectives: first, to embed a representation of each word based on its semantic meaning and, second, to store the semantic temporal context in the recurrent layers. The remaining component is the multimodal (φ) component, which is in charge of processing both CNN and RNN outputs and generating the output of the model.

As discussed in the previous section, we have developed an architecture composed of two M-RNNs (see Figure 2A,B). The first one, the M-RNN_*A*_, predicts the *attributes* that are incorporated as input to the second one, the AM-RNN, which performs the IUPAC name prediction. Although both AM-RNN and M-RNN_*A*_ are based on the standard M-RNN,^43^ we introduce substantial modifications in each component. In Figure 2A,B, we show the inputs for each component. The input of the CNN component is a stack of 10 AFM images, whereas the input of the multimodal component φ consists of a concatenation of the outputs of the CNN and RNN components. A detailed description of the structure and role of the CNN, RNN, and multimodal φ components can be found in Figure S1 and Sections S2 and S3.

To explicitly define the inputs of the M-RNN components, it is worth recalling that an M-RNN processes time series, so it will perform a prediction (*attribute* or *term*) at each time step. Let us start by defining the inputs of the RNN component of the M-RNN_*A*_. We encode the *attributes* of the model by assigning integer numbers (from 1 to 100) to each *attribute*. The input of RNN is a vector of fixed size 19, the maximum number of different attributes in the names of the molecules in QUAM–AFM (17) plus the *startseq* and *endseq* tokens. In the first step, it will contain *S*_0,M-RNN_*A*__ = *startseq* to provide the model with the information that a new prediction starts. This input is padded with zeros until we obtain a length of 19 (see Figure 2A,C,E) and then processed by the RNN component while the stack of AFM images are processed by the CNN component, each of them encoding the respective input into a vector. The two resulting vectors are used to feed the multimodal component φ, where they are concatenated and processed in a series of fully connected layers to finally produce a vector of probabilities (see Figures S1 and S2 for details on the RNN and φ layers). In this way the prediction at each time step corresponds to the most likely *attribute Y*^*A*^_1_ which replaces the padding zero of the corresponding position in the input sequence of the RNN component in the next time step. This process is repeated until the *endseq* token is predicted, which breaks the loop. That is, for a given time step *t*, we feed the RNN component of M-RNN_*A*_ with the input (*S*_0_, *Y*_1_^*A*^, ..., *Y*_*t*–1_^*A*^) that concatenates the *starseq* token *S*_0_ with all the predictions already performed in previous time steps, which is padded with zeros until we obtain a length of 19 (see Figure 2E for the example of *t* = 4 in the identification of perylene-1,12-diol molecule). Once the model has already predicted the *N*_*A*_*attributes*, it has to break the loop, so its last prediction must be the *endseq* token (see Figure 2C).

Once the prediction of the *attributes* has finished, the AM-RNN starts to operate in order to predict the IUPAC name of the molecule. For the input of the RNN component and the prediction flow, we follow the same reasoning applied to M-RNN_*A*_, replacing *S*_0,M-RNN_*A*__ by *S*_0,AM-RNN_ = (*Y*_1_^*A*^, .., *Y*_18_^*A*^, *startseq*) (Figure 2B). Each RNN input is a vector of 76 components, arising from the concatenation of 18 *attributes* (*Y*_1_^*A*^, .., *Y*_18_^*A*^) ∼ (*A*_1_, ..., *A*_18_) (padded if necessary) with the *startseq* token and the predictions performed at each previous time step, (*Y*_1_, ..., *Y*_*t*–1_), padded until we obtain a vector with length 57—the maximum number of terms in the decomposition of the IUPAC names in QUAM–AFM (see Figure 2C,F). Similarly as in the M-RNN_*A*_, the semantic input is processed by the RNN component while the AFM image stack is processed by the CNN, encoding the respective input into a vector. The multimodal component φ processes the CNN output *v*, concatenates the result with the output of the RNN, and processes this combined result producing a vector of probabilities as output of the network (see Figures S1 and S2 for details). The position of the larger component in the vector provides us with the prediction of the new *term Y*_*t*_. The process stops when the *endseq* token is predicted (see Figure 2C).

## Supporting Information Available

Detailed information on IUPAC tokenization, description of the M-RNNA and AM-RNN models, model training, influence of the molecular torsion in the model performance, experimental tests, and learning of syntactical relations shown by the embedding analysis (PDF)

## Abbreviations

DFT: density functional theory
AFM: atomic force microscopy
GUI: graphic user interface
PPM: probe particle model
FDBM: full density-based model
DP: deep learning
AI: artificial intelligence
BLEU: Bilingual Evaluation Understudy
RNN: recurrent neural network
CNN: convolutional neural network
