# Supplementary material for: Molecular Identification from AFM Images Using the IUPAC Nomenclature and Attribute Multimodal Recurrent Neural Networks
Source: ACS Appl Mater Interfaces. 2023 May 1;15(18):22692–704. doi: 10.1021/acsami.3c01550 (PMC10176476; doi:10.1021/acsami.3c01550)
Supplement: Supplementary file 1 — am3c01550_si_001.pdf [file am3c01550_si_001.pdf]

Supporting Information for Molecular Identification from AFM images using the IUPAC Nomenclature and Attribute Multimodal Recurrent Neural Networks

Jaime Carracedo-Cosme,[1,2] Carlos Romero-Muñiz,[3]
Pablo Pou,[2,4] Rubén Pérez,[2,4,*]

[1]Quasar Science Resources S.L., Camino de las Ceudas 2, E-28232 Las Rozas de Madrid, Spain
[2]Departamento de Física Teórica de la Materia Condensada,
Universidad Autónoma de Madrid, E-28049 Spain
[3]Departamento de Física Aplicada I, Universidad de Sevilla, E-41012, Seville, Spain
[4]Condensed Matter Physics Center (IFIMAC),
Universidad Autónoma de Madrid, E-28049 Madrid, Spain

* ruben.perez@uam.es

Contents

S1 IUPAC tokenization 3

S2 M-RNN$_A$ and AM-RNN models: A layer description 5

S3 Model learning 8

S3.1 Molecular Classes for Transfer Learning 8

S3.2 Pre–training the CNN component 9

S3.3 M–RNN and AM–RNN optimization 10

S3.4 M–RNN and AM–RNN training 11

S4 Influence of the molecular torsion in the model performance: gas–phase versus flat configurations 13

S5 Experimental test 16

S6 Embedding 18

References and Notes 21

S1 IUPAC tokenization

In order to tokenize the IUPAC nomenclature we selected a set of terms consisting of prefixes, suffixes, numbers, etc., whose combinations generates the 686,000 IUPAC names corresponding with the molecules belonging to Quasar Science Resources - Universidad Autónoma de Madrid - Atomic Force Microscopy Image Dataset (QUAM-AFM).[1] Deep learning models require large datasets in which each target is repeated many times to be learned during the training. We have analysed the number of times that each term appears in QUAM-AFM and removed those terms that are repeated less than 100 times. This reduces the total number of terms considered to a total of 199, shown in table S1. Consequently, we have discarded molecules whose decomposed IUPAC name contains any of these terms. Similarly, extremely long IUPAC names have very little representation in QUAM-AFM, so we also discard molecules whose IUPAC name decomposes into more than 57 terms. In this way, we have slightly reduced the set of available inputs to a total of 678.000 molecules.

acen	acet	acid	acr	alde	alen	amate	amide	amido	amim
amine	amino	amo	ane	ani	anil	ano	anone	anthr	ate
ato	aza	aze	azi	azido	azin	azo	azol	benz	brom
but	carb	chlor	chr	cyclo	ene	eno	eth	fluor	form
furan	furo	hyde	hydr	ic	ida	ide	idin	idine	ido
imid	imidin	imine	imino	ind	ine	ino	iod	iso	itrile
ium	meth	mine	naphth	nitr	nium	nyl	oate	oic	ol
ole	oli	olin	oline	olo	om	one	oso	oxo	oxol
oxy	oxyl	oyl	phen	phth	prop	pter	purin	pyr	pyrrol
quin	sulf	thi	tri	urea	yl	ylid	yridin	zin	zine
dodeca	undeca	lambda	phosph	cinnam	xanth	porph	coron	deca	guan
hept	amic	pent	enal	octa	anal	nona	mido	nida	azet
tetr	ulen	anol	hypo	pine	ysen	anth	tere	acyl	yrin
inin	tris	pino	mid	hex	nia	bis	per	nio	pin
ite	rin	alo	en	di	11	yn	an	cy	de
15	10	13	14	12	in	18	21	az	al
bi	et	ep	id	ox	il	or	16	17	on
(	6	)	-	[	a	]	N	’	1
7	4	5	3	9	2	8	H		,
o	b	e	c	C	d	O	g	f	

Table S1: Table of terms for International Union of Pure and Applied Chemistry (IUPAC) decomposition. The elements above the bold line are the subset of 100 attributes considered. The grey cell does not correspond to any term, it has been coloured in order to distinguish it from the term that spells an empty space between two words.

As explained in the main text, we combine two different Multimodal Recurrent Neural Network (M-RNN)s to achieve molecular identification. The first network, named Multimodal Recurrent Neural Network for attribute prediction (M-RNN$_A$), uses as input the stack of Atomic Force Microscopy (AFM) images and its aim is to extract the *attributes*, a 100-element subset of the terms which are mainly used to designate functional groups and not positions (see table S1). The second network, Atribute Multimodal Recurrent Neural Network (AM-RNN), consists of an adaptation of the M-RNN[2] for the IUPAC nomenclature prediction, where we add an input of semantic attributes predicted by the M-RNN$_A$ (see section S2 for details). This strategy differs from the concept of *attention*,[3,4,5] implemented in other Natural Languaje Procesing (NLP) problems, and that has led to the development of the transformers.[6,7,8,9] In this case we assume that the relationship between each IUPAC name and each molecule is biunivocal, (although there are some exceptions we suppose that the names of each compound have been entered under the same rules in Pubchem). This approach makes the problem posed different from most of the challenges faced in image captioning where there are multiple descriptions for the same image. Therefore, a prior identification of the main functional groups, not only releases the Convolutional Neural Network (CNN) component of the AM-RNN from the task of identifying these moieties, but, more importantly, almost halves the number of possible predictions of the AM-RNN: By feeding the AM-RNN with the attributes that are present in the IUPAC name (predicted by the M-RNN$_A$), we are also effectively excluding the large number of them that do not form part of it. This strategy improves significantly its performance.

S2 M-RNN$_A$ and AM-RNN models: A layer description

As previously mentioned, the architecture developed for molecular identification with the IUPAC nomenclature is composed of two models, M-RNN$_A$ and AM-RNN, (see Figure 2 of the main text). Each one is composed by a CNN, a RNN and a multimodal componet φ. Figure S1 displays the type of layers that constitute each component of both M-RNN$_A$ and AM-RNN. The architecture of the CNN component is identical in both models, while RNN and φ share the same structure and, except for the RNN layer, the same type of layers with different number of units (e.g. kernels in convolutional layers, vector length in fully connected layers, etc) according to the specific purpose of the model. Figure S2 provides the details, highlighting the

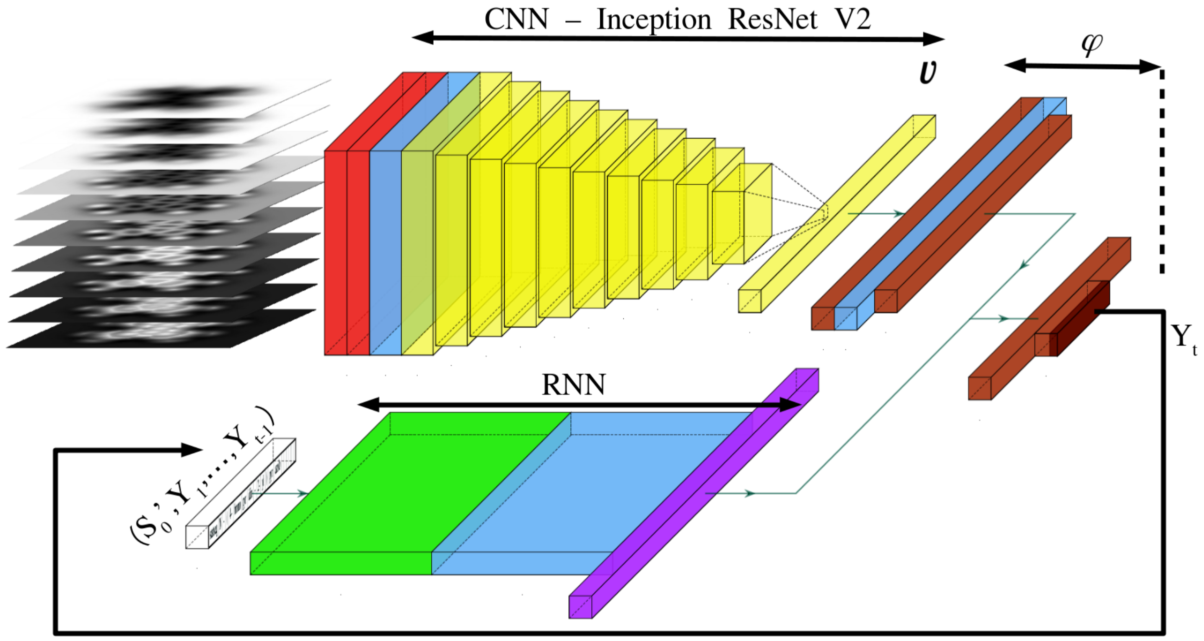

Figure S1: Graphical layer representation of M-RNN$_A$ and AM-RNN with the layers that constitute their three components, CNN, RNN and φ. The CNN component follows the Inception ResNet V2 model, where the first 2D–convolutional layers have been replaced by two 3D convolutional layers (to process the image stack, shown in red), followed by a dropout layer (blue). The yellow blocks are just a pictorial representation of each block of the original Inception ResNet V2 model. Notice that the last fully connected layer of the model, which is specific for the original classification task, has been removed, obtaining an output vector (v) with length 1539. The RNN and φ components include embedding (green), dropout (blue), fully connected (brown) and recurrent (purple) layers. The purple box represents a Gated Recurrent Unit (GRU) layer in M-RNN$_A$, whereas in the AM-RNN it represents a Long Short-Term Memory (LSTM) layer. S'_0 represents the input for the RNN component in the first time step: the *startseq* token in the M-RNN$_A$ and the concatenation of the *attributes* with the *starseq* token in AM-RNN. The subsequent inputs include the *attributes* (*terms*) predicted in previous time steps by M-RNN$_A$ (AM-RNN). Although both M-RNN$_A$ and AM-RNN have the same structure, each layer of RNN and φ components has different dimensions (see Figure S2 for a detailed description).

differences between the layers of the RNN and φ components of the two models.

The CNN component consists of a modification of the Inception ResNet V2 model,[10] identical for both M-RNN$_A$ and AM-RNN. This well–known model has been developed to be applied to 2D images whereas in our case we process 3D maps (stacks of 10 AFM images with various tip–sample distances). Therefore we have replaced the first 2D–convolutional layers in Inception ResNet V2 by two 3D convolutional layers, each one with 32 filters, (3,3,3) kernel size and (2,1,1) strides, followed by a dropout layer. We have verified that this dropout layer is essential for the model to generalize to different images, such as the experimental ones. In addition, we have removed the last fully connected layer of the model, which is specific for the original classification task, obtaining an output vector (v) with length 1539.

The goal of the RNN component is to use sequential data to add new *terms* to the formulation. To this end, the architecture of this component is developed according to two key

Model	Component	Operator	Units	Activation	Connections
M-RNN$_A$	RNN	Input$_{RNN}$	19		
		Embedding	32		Input$_{RNN}$
		Dropout$_1$	0.2		Embedding
		GRU	128		Dropout$_1$
	ψ	FC$_1$	1024	ReLU	v
		Dropout$_2$	0.2		FC$_1$
		FC$_2$	512	ReLU	Dropout$_2$
		Concat			FC$_2$, GRU
		FC$_3$	256	ReLU	Concat
		FC$_4$	103	Softmax	FC$_3$
AM-RNN	RNN	Input$_{RNN}$	76		
		Embedding	32		Input$_{RNN}$
		Dropout$_1$	0.2		Embedding
		LSTM	256		Dropout$_1$
	ψ	FC$_1$	1024	ReLU	v
		Dropout$_2$	0.2		FC$_1$
		FC$_2$	512	ReLU	Dropout$_2$
		Concat			FC$_2$, GRU
		FC$_3$	256	ReLU	Concat
		FC$_4$	202	Softmax	FC$_3$

Figure S2: Layer–by–layer details of the RNN and φ components integrated in M-RNN$_A$ and AM-RNN. v denotes the output vector of the CNN component. The layers are the same for both models, except for the recurrent one, a GRU in M-RNN$_A$ (attribute prediction) and an LSTM in AM-RNN (term prediction).

objectives: Firstly, to embed a representation of each *term* based on its semantic meaning and, secondly, to store the semantic temporal context in the recurrent layers. To perform the representation of each *term* in a vector space, the RNN component has an embedding layer that is able to capture relationships between *terms* (see section S6 for a more detailed analysis). The embedding layer is followed by a dropout layer that acts as a regularizer and finally the data goes through a recurrent layer which, with the temporal context generated by all the predictions made in previous time steps, makes a proposal of predictions that is processed by the multimodal component (see fig. S1). The recurrent layer is a GRU in M-RNN$_A$ (attribute prediction) and an LSTM in AM-RNN (term prediction). The multimodal component φ first processes the CNN output v in two fully connected layers with a dropout between them. Subsequently, this output is concatenated with the output of the RNN (see fig. S1). Finally, the result of the concatenation feeds two fully connected layers, the first one activated with Rectified Linear Unit Activation Function (ReLU) activation function whereas the second one is activated with Soft Approximation of Max (Softmax) activation function, that converts the outputs from the layer into a vector with components that represent probabilities that sum to one.

S3 Model learning

In principle, it could be possible to train the CNN in conjunction with the RNN in an end–to–end process. However, this would require a massive amount of computational time (more than a year even with the good GPU resources available to us). Moreover, as each of the components has a very different depth, the weight of the backpropagation values would overfit the RNN component. This behavior has been discussed in depth in the literature (see, for example, ref.[11]). To avoid it, we train each component of the models in different stages, fixing the weights of the CNN and RNN alternatively while training the rest of the model.

In the first stage, both CNN and RNN components are initialised with random weights, so if we fix the weights of the CNN while training the RNN, the CNN would perform a random representation of the input image stack, and consequently, the weights of the RNN component would be updated under random rules. An analogous reasoning can be applied for the reverse case, in which we would fix the weights of the RNN and train the CNN. We solve this issue initialising the CNN component with pre–trained weights. To determine this weights, we perform a classification with the CNN based on classes of molecules that shared the same chemical composition (number of different chemical species and number of atoms for each specie, excluding the H atoms), as described in detailed in section S3.1.

S3.1 Molecular Classes for Transfer Learning

The classification of AFM images defining the model output as each individual molecule is an impossible task because QUAM-AFM does not include enough images of each particular structure and has an excessive number of molecules (classes). Thus, we simplify the problem by grouping molecules based on their chemical composition. Hence, we define the class of a molecule by the type of atomic species that it contains and the number of repeated atoms of each of these species. To obtain a representative number of images of each class, we exclude the hydrogens from the species list (see fig. S3), so that molecules with completely different structures such as pyrazine, pyridazine, but-2-enedinitrile or butanedinitrile belong to the same class (C_4N_2). This results in a total of 2339 classes for the molecule structures considered in QUAM-AFM.

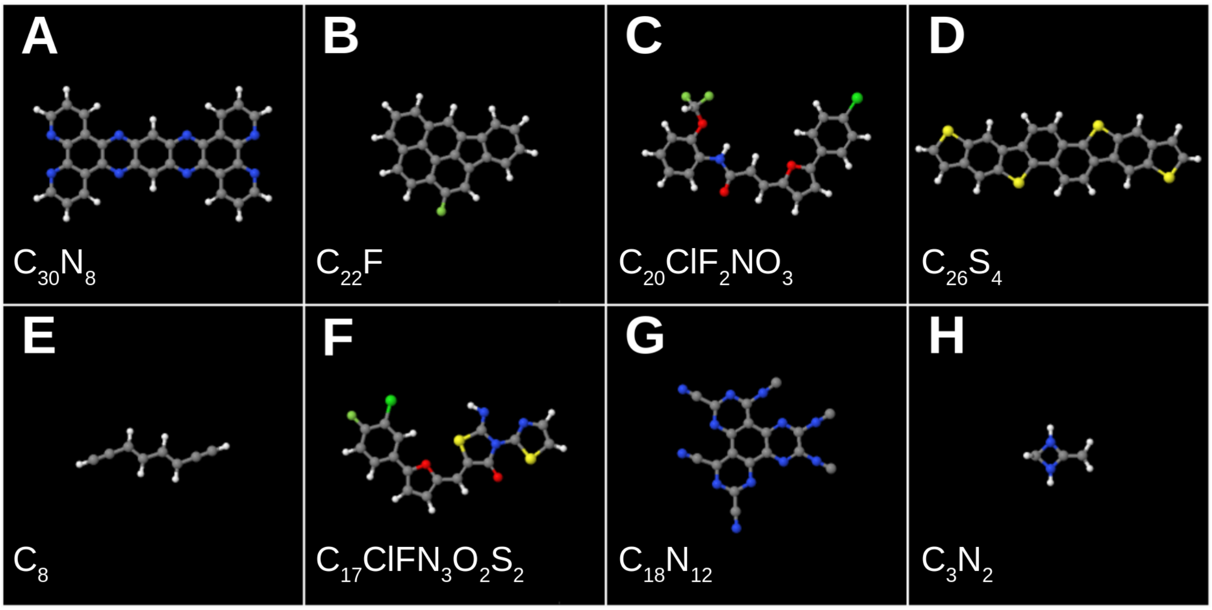

Figure S3: Atomic structures belonging to different classes for classification according to their chemical species.

S3.2 Pre–training the CNN component

As mentioned above, it is necessary to pre-train the CNN component with a classification that groups the molecules in the QUAM-AFM dataset in classes describing its chemical composition, irrespective of their structure. We perform this classification with the same Inception ResNet V2 model[10] that we used for the CNN element in the two M-RNNs . To this end, we replace the first convolutional layer by two 3D convolutional layers followed by a dropout layer and, instead of removing the output layer as described in the main text for the NLP target, we modify its number of units to 2339, corresponding to the number of classes defined in section S3.1.

In each epoch, we select a single combination of simulation parameters from those included in the QUAM-AFM dataset[1] for each molecule. Therefore, the model receives inputs of the same molecules but different image stacks at each epoch. The Image Data Generator (IDG) includes rotations, zoom, shear and vertical and horizontal shifts (translations). The goal of these operations is two-fold: to incorporate translational and rotational invariance, and to take into account deformations in the images (due to slight asymmetries of the CO tip or to experimental noise), as discussed in ref. 12. The values selected for the distortions are randomly chosen in the following ranges: [1,360]–degree rotations, ± 0.15 zoom range, ± 0.1 shear range, and ± 0.1

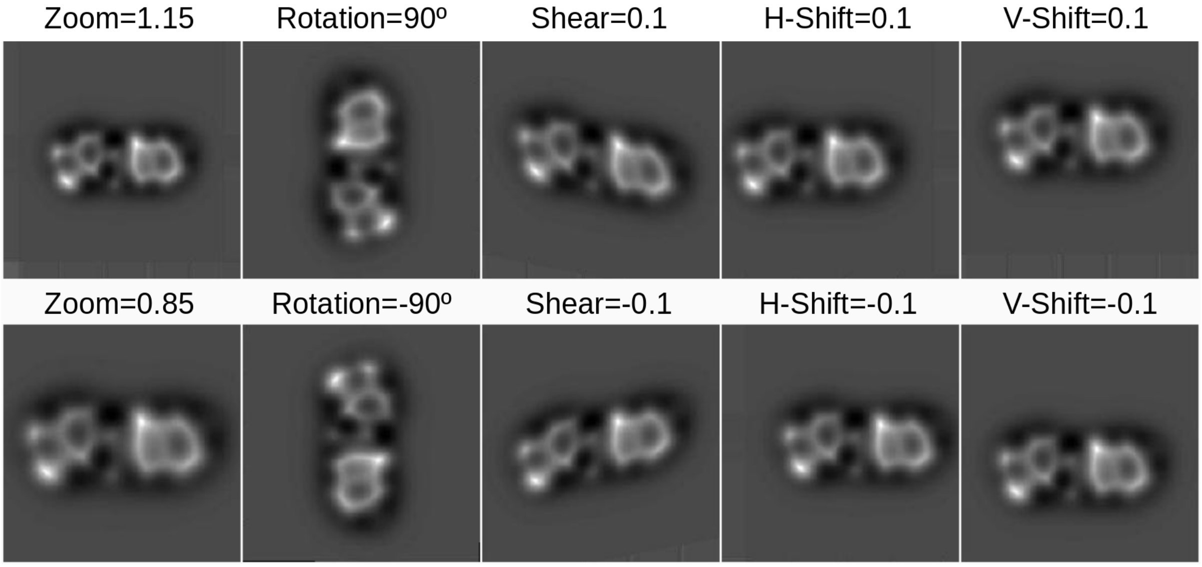

Figure S4: Results of applying the IDG to the same AFM image of a N-(5-amino-4-methylpyridin-2-yl)-6-fluoro-1-benzothiophene-2-carboxamide molecule.

both vertical and horizontal shift range, as illustrated in fig. S4. When a molecule is rotated or moved during an AFM experiment, all resulting images show similar variations, so we apply the same deformation parameters to the ten images that compose each stack. This training (that includes complete rotations and 10% shifts) ensures that the model is robust with respect to rotational and translational operations and, coupled with the dropout layer in the CNN component, also to soft variations of the inputs that are present in experimental images.

We train the CNN minimizing the error of the Negative Log Likelihood loss function with the Adaptive Moment estimator (Adam) optimizer.[13] To speed up the convergence, we apply a batch–normalization,[10,14] setting the mean to zero and the variance to one in the input layers.[15,16] We found that this normalization not only makes the training faster but also improves the classification results, reaching an accuracy of 0.97 in the test prediction.

S3.3 M–RNN and AM–RNN optimization

Because of the analogies between M-RNN$_A$ and AM-RNN, their loss functions and trainings are similar, hence we define the optimization problem for both at the same time with the notation used in the main text. The function to be maximized is the probability of obtaining a correct sentence $S = (S_1, ..., S_N)$ given an input (I, S'_0):

$$\theta^* = arg\max_{\theta} \sum_{(I,S)} \log p(S|I, S'_0; \theta) \tag{1}$$

where θ represents the model parameters, and I is the 3D image stack. Note that the prediction of the IUPAC nomenclature, according to the decomposition performed on a set of terms, must depend on the predictions already performed, i.e. the prediction must take into account previously predicted terms in order to have semantic meaning, so it is a time series. During the training, we feed the model at each time step t with the target of previous time steps $(S_0', S_1, ..., S_{t-1})$ and not with the predictions performed $(Y_1, ..., Y_{t-1})$, which in some cases are wrong. We refer to each input of the model at the time step t as the pair $(S_0', S_1..., S_{t-1}; I)$. Note that, in this way, the final prediction length of each molecule depends on t. Thus, the prediction of S depends on the prediction of each specific term, which in turn depends not only on I but also on all the predictions performed in previous time steps. Since the model predicts a single term of the sequence at each time step, it is natural to apply the chain rule to model the joint probability over the sequential *terms*. Hence, the probability of obtaining a correct prediction for the complete sequence is described by the sum of the logarithmic probabilities over the terms. Therefore, the maximum log–likelihood function is as follows:

$$L(S, I) = \sum_{t=1}^{N} \log p(S_t | I, S_0', S_1, ..., S_{t-1}; \theta). \quad (2)$$

As the deep learning optimization techniques consist of searching for a minimum rather than a maximum, the loss function is described by the sum of the negative log–likelihood:

$$L(S, I) = -\sum_{t=1}^{N} \log p(S_t | I, S_0', S_1, ..., S_{t-1}; \theta). \quad (3)$$

S3.4 M–RNN and AM–RNN training

The training of both M-RNN$_A$ and AM-RNN proceeds in three stages in which the weights of the CNN and RNN components are fixed (non-trainable) alternatively. In the first stage, the model initialises all its weights randomly except those of the CNN component, which are pre–trained with the chemical classification explained in section S3.2, and focuses on the training of the RNN. Although specialised in a different classification, the CNN component output already represents high–level features of each input stack. The model is then fed with the input (I, S). In the same way as we do for the CNN pre-training described in section S3.2, a random combination of the simulation parameters described in[1] is selected for each input at each epoch. Thus,

M-RNN$_A$

Stage 1. CNN fixed			Stage 2. RNN fixed			Stage 3. CNN fixed		
Lr	Epochs	Batch	Lr	Epochs	Batch	Lr	Epochs	Batch
10^{-3}	5	64	10^{-3}	-	32	10^{-3}	20	64
10^{-4}	3		10^{-4}	6		10^{-4}	10	

AM-RNN

Stage 1. CNN fixed			Stage 2. RNN fixed			Stage 3. CNN fixed		
Lr	Epochs	Batch	Lr	Epochs	Batch	Lr	Epochs	Batch
10^{-3}	10	64	10^{-3}	-	32	10^{-3}	40	64
10^{-4}	5		10^{-4}	8		10^{-4}	30	

Figure S5: Details of the training scheme for each stage of each of the models. Lr is a short for learning rate.

although the CNN's weights are fixed, the high–level representation of each structure is different in each epoch. This selection, coupled with the dropout layer of the φ component, ensures that the RNN component does not overfit for specific representations of the input images.

The aim of the second stage is to specialise the weights of the CNN component in the semantic prediction. To this end, the weights of the RNN component are fixed and the IDG described in section S3.2 is applied to the input stack. Furthermore, the selection of simulation parameters is randomly chosen for each input I. In this stage the prediction is performed for a single time step of each pair (S, I). After completion of this second stage, the CNN component provides specific details for the IUPAC formulation rather than to the chemical classification. Finally, the third training stage repeats the process of the first one, fixing the weights of the CNN. Further details for the training at each stage (number of epochs, batch size, learning rate) are shown in fig. S5.

S4 Influence of the molecular torsion in the model performance: gas–phase versus flat configurations

HR-AFM shows an outstanding lateral resolution for quasi-planar molecules, but it is more limited to discern correctly molecules with a significant torsion. This is due to the nature of the contrast and the probe flexibility. The exponential behavior of the Pauli repulsion with respect to the tip-sample distance makes this contribution the most relevant contrast source. Thus, each atom in the sample will behave as an umbrella of around 2-3 Å veiling any other electronic charge density below this region (except some cases where deformations in the charge density may occur). Furthermore, the CO molecule will tilt under this repulsion and will block the access of the probe to the region underneath.

Previous works[17] suggest that it is difficult to retrieve information from parts of the molecule that are located more than 1.5 Å below the topmost atoms. According to this, we expect our algorithm to provide a poorer performance when finding out the IUPAC names of molecules showing significant out-of-plane distortions. In order to quantify this, we have carried out some tests to directly show how the model accuracy improves for planar structures. We have selected four molecules showing a torsion of about 1.8 Å and recalculated them in a forced planar configuration. These planar configurations have been determined by DFT calculations where we fix the z-position of all the atoms in the molecule and let them move in the xy plane in order to reach the ground state configuration compatible with this constraint. If we feed the algorithm with the image stacks of the planar configuration the Bilingual Evaluation Understudy (BLEU) 4-gram score increases significantly in all cases, even when the chemical nature of the molecule makes the recognition by our model more difficult. Figure S6 shows two molecules where the prediction for the non planar failed but where we obtain a highly accurate result (an almost perfect match in the cases shown) for the planar configurations, with only an error in the misplacement of one of the functional groups. Figure S7 shows the result for two other molecules that contain functional groups that are difficult to discern such as fluorine atoms, easily mistakable with other terminations like diketones which may display faint AFM features. Although the BLEU 4-gram for the planar configuration is not high there is a clear improvement compared to the non–planar cases.

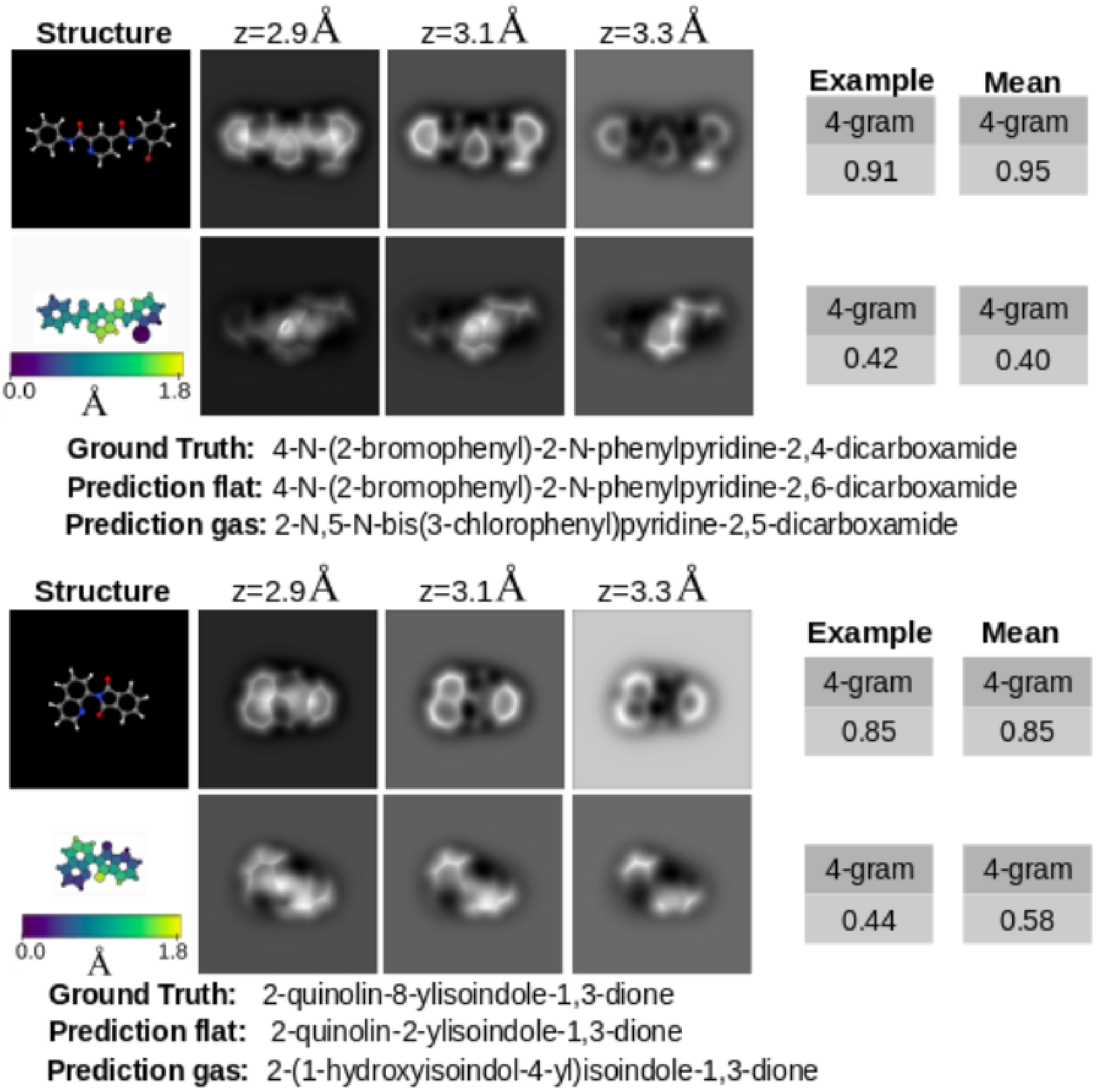

Figure S6: Comparison of the model predictions for the gas–phase and the forced planar configuration of a representative molecule, whose ball–and–stick depiction and height map are shown on the left. The upper AFM images correspond to the simulation with the structure in a completely planar configuration while the lower ones correspond to the images for the gas–phase structure. To the right of the AFM images is the 4–gram score corresponding to the prediction shown (flat and gas–phase structure, respectively). Further to the right is the average of the 4–gram scores obtained for images generated with the 24 combinations of operational parameters considered in QUAM-AFM. The prediction example shown is the one that scored closest to the mean.

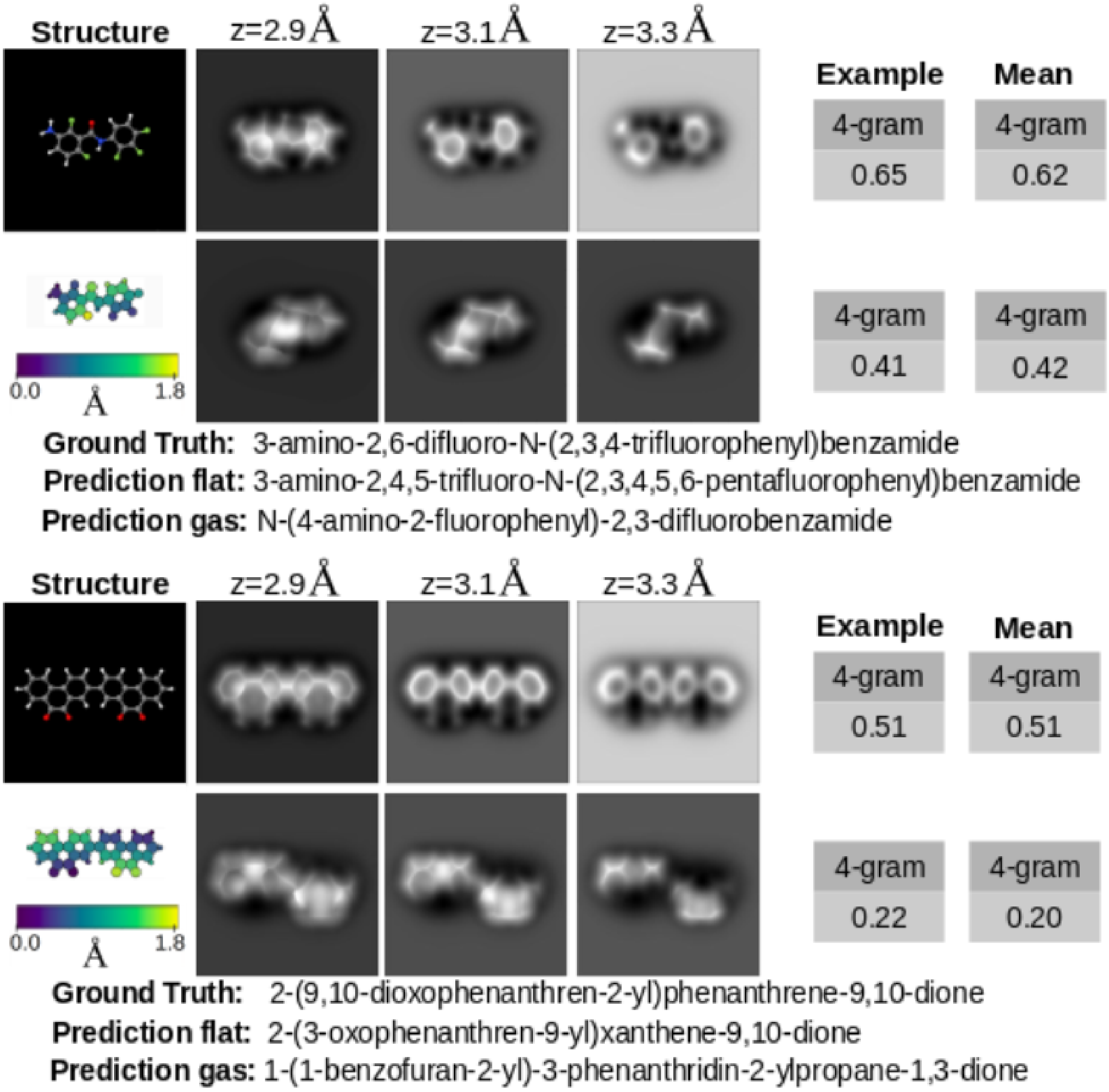

Figure S7: As fig. S6 for two molecules that include chemical chemical groups, like F atoms (top) and diketones (bottom), that display faint AFM features and thus are more difficult to recognise by the model.

S5 Experimental test

We have performed a test with 3D stacks of experimental AFM images for dibenzothiophene and 2-iodotriphenylene (see fig. S8). In the first case, despite the strong noise in the images and the white lines crossing the images diagonally, we obtain a perfect prediction. In the second case, with images covering a tip-sample distance range of 72 pm (smaller that the 100 pm range used for the training), the prediction performed by the model is “2iodtriphenylene”, which is very close to the ground truth, just missing a hyphen but providing all the relevant chemical

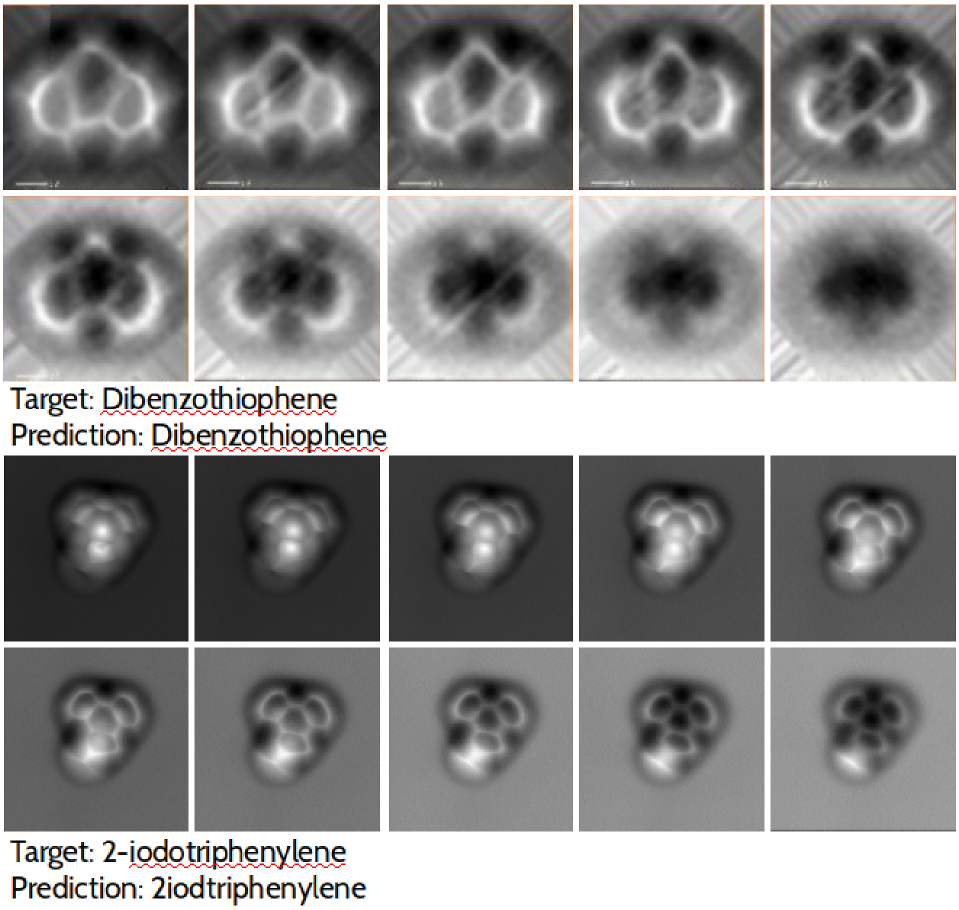

Figure S8: Experimental images used to test the molecular identification with our model. The top set of images corresponds to the 10 images of dibenzothiophene taken at 10 different tip-sample distances (covering a height range of 100pm) in ref.[18] (reproduced courtesy of the American Chemical Society, ACS), using a bias voltage $V = 40$ mV and a tip oscillation amplitude of approximately 50 pm. Images were aligned and Laplace filtered in the original publication to highlight the molecular structure while maintaining the feature shapes as well as possible. The bottom set corresponds to 10 constant–height frequency shift images of 2-iodotriphenylene, covering a height range of 72 pm (with a height variation of 8 pm between two consecutive images), published in the supplementary material of ref.[19] (reproduced courtesy of the American Physical Society, APS). A bias voltage $V = -0.57$ mV and an oscillation amplitude of $A = 52$ pm have been used for the measurements.

information. Notice that, in both cases, experimental images have been scaled close to the size of the unit cell used in QUAM-AFM before feeding the model. Without this scaling, the score in the 4-gram evaluation falls dramatically for both cases, suggesting that the typical sizes of chemical moieties learned during the training are an important component in the success of the identification process. This sensitivity to variations in image sizes can possibly be reduced by increasing the zoom range of $\pm 15\%$ applied in our data augmentation during the training.

S6 Embedding

The weights of the embeddings of the RNN components of models applied to image captioning[20,21] are usually pretrained with Word Embeding to represent the semantic high-level features in a vectorial space. The usefulness of this technique lies in the representation of words in a vector space, in which words with similar semantic meaning are represented close together, as shown in fig. S9. In this way, the models manage to use synonyms providing versatility and improving its expressive capacity. The versatility of languages to express the same idea in different ways is further exploited in image captioning providing the same input image with different annotation outputs. Then those models succeed in learning that different words have

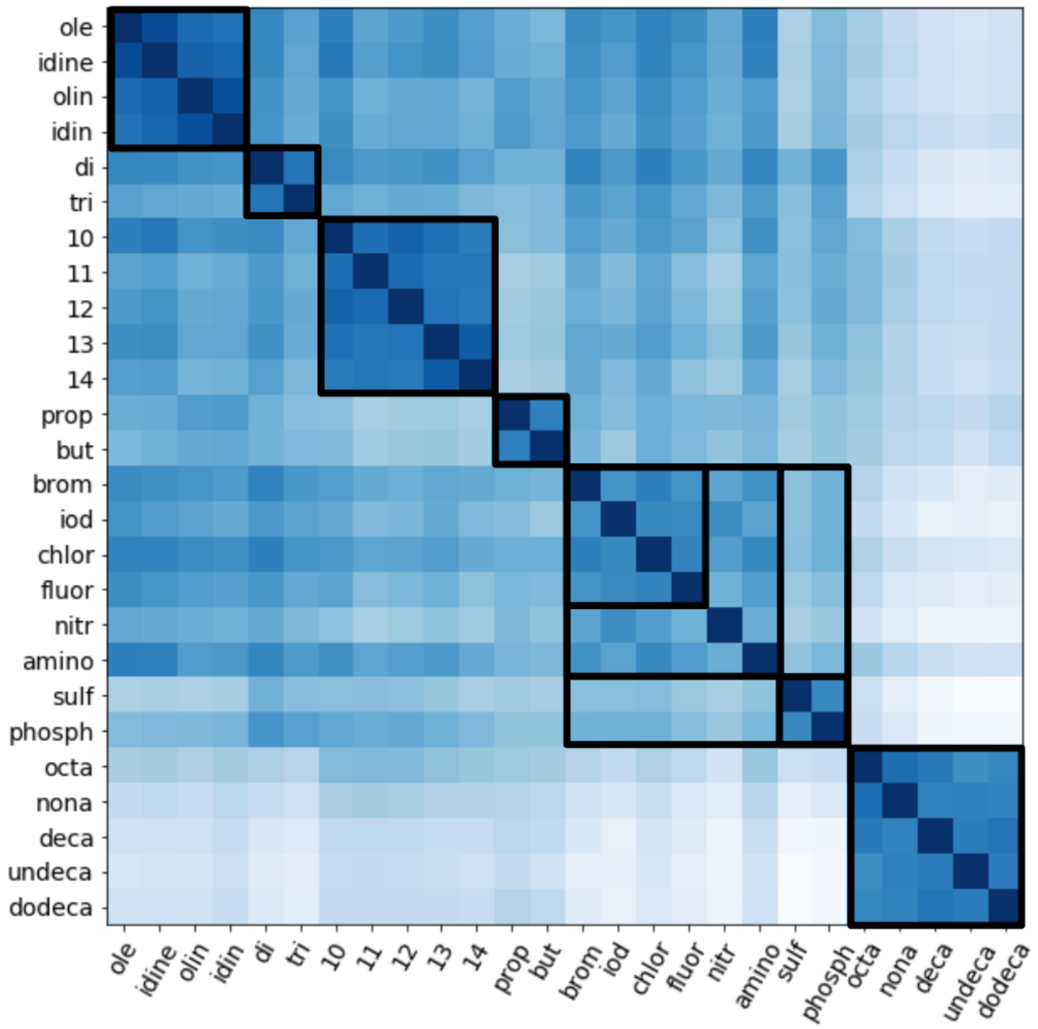

Figure S9: Distances in the L2 sense between some of the terms in the AM-RNN embedding layer. The higher intensity of blue corresponds to smaller distances while the lighter ones correspond to longer distances. The distance from a term to itself is zero, so it matches the darkest blue.

similar meaning.

However, there are no trivial synonyms in our case, due to the close relationship between the IUPAC names assigned to the functional groups in a molecule. Thus, any change in the output sequence will result likely in an error, as a different molecule would be predicted. Additionally, there is no freely available pre-trained Word Embeding for the IUPAC nomenclature, and therefore all weights of the RNN component are initialised randomly. Consequently, we train the embedding layer with the rest of the RNN component (stages 1 and 3 described in section S3.4). Here we find that, although the IUPAC nomenclature does not use synonyms, the representations made in this vector space have certain semantic relationships. To check this, we define the distance in the L2 sense and calculate the distances among the terms (of the AM-RNN). We find, for example, that the terms represented closest to *brom* are *chlor, fluor* and *iod*. In addition to the terms associated with halogens, those designating numbers are also represented in close proximity. It is also noteworthy that the terms closest to *nona* are *octa, deca, undeca* and *dodeca*, as shown in fig. S9. It should be recalled that each of the terms is represented by a number to feed the network, so the model is not learning lexical relations but syntactic relations.

References and Notes

1. J. Carracedo-Cosme, C. Romero-Muñiz, P. Pou, R. Pérez, *J. Chem. Inf. Model.* **62**, 1214 (2022).

2. J. Mao, *et al.*, *arXiv preprint arXiv:1412.6632* (2014).

3. D. Bahdanau, K. Cho, Y. Bengio, *arXiv preprint arXiv:1409.0473* (2014).

4. A. P. Parikh, O. Täckström, D. Das, J. Uszkoreit, *arXiv preprint arXiv:1606.01933* (2016).

5. C. Raffel, M.-T. Luong, P. J. Liu, R. J. Weiss, D. Eck, *International Conference on Machine Learning* (PMLR, 2017), pp. 2837–2846.

6. A. Vaswani, *et al.*, *Proceedings of the 31st Annual Conference on Neural Information Processing Systems (NIPS)* (Long Beach, CA, USA, 2017), pp. 5998–6008.

7. J. Devlin, M.-W. Chang, K. Lee, K. Toutanova, *arXiv preprint arXiv:1810.04805* (2018).

8. T. B. Brown, *et al.*, *arXiv preprint arXiv:2005.14165* (2020).

9. Y. Pan, T. Yao, Y. Li, T. Mei, *Proc. Comput. Vision. Pattern Recognit. (CVPR)* (IEEE Computer Society Press, Piscataway, NJ, USA, 2020), pp. 10971–10980.

10. C. Szegedy, S. Ioffe, V. Vanhoucke, A. A. Alemi, *31rd Proc. AAAI Conf. on Artificial Intelligence* (AAAI Press, Palo Alto, CA, USA, 2017), p. 4278–4284.

11. J. Gu, J. Cai, G. Wang, T. Chen, *32rd Proc. AAAI Conf. on Artificial Intelligence* (AAAI Press, Palo Alto, CA, USA, 2018).

12. J. Carracedo-Cosme, C. Romero-Muñiz, R. Pérez, *Nanomaterials* **11**, 1658 (2021).

13. F. Chollet, *Deep Learning with Python*, vol. 1 of *10* (MANNING, Shelter Island, NY 11964, 2015), first edn.

14. P. Y. Simard, Y. A. LeCun, J. S. Denker, B. Victorri, *Neural networks: tricks of the trade* (Springer-Verlag Berlin Heidelberg, 1998), pp. 239–274.

15. S. Ioffe, C. Szegedy, *32th Int. Conf. Mach. Learn. (ICML)* (JMLR.org, 2015), vol. 37, p. 448–456.

16. Y. LeCun, L. Bottou, Y. Bengio, P. Haffner, *Proc. IEEE* **86**, 2278 (1998).

17. B. Alldritt, *et al.*, *Sci. Adv.* **6**, eaay6913 (2020).

18. P. Zahl, Y. Zhang, *Energy Fuels* **33**, 4775 (2019).

19. D. Martin-Jimenez, *et al.*, *Phys. Rev. Lett.* **122**, 196101 (2019).

20. Q. You, H. Jin, Z. Wang, C. Fang, J. Luo, *Proc. Comput. Vision Pattern Recognit. (CVPR)* (IEEE Computer Society Press, Piscataway, NJ, USA, 2016), pp. 4651–4659.

21. O. Vinyals, A. Toshev, S. Bengio, D. Erhan, *Proc. Comput. Vision Pattern Recognit. (CVPR)* (IEEE Computer Society Press, Piscataway, NJ, USA, 2015), pp. 3156–3164.
